# Rizedisben in Minimally Invasive Surgery: A Nonrandomized Clinical Trial

**DOI:** 10.1001/jamasurg.2025.1987

**Published:** 2025-07-02

**Authors:** Samuel A. Gold, Maria M. Pere, Melissa Assel, Alexander D. Doudt, Trey D. Durdin, Andrew W. Silagy, Lucas W. Dean, Pedro Recabal, Erica Levine, Alan Burke, Govind Ragupathi, Mohammad R. Marzabadi, Zhong-Ke Yao, Guangbin Yang, Guangli Yang, Ouathek Ouerfelli, Melissa McCarter, Xi Chen, Efstathia Tzatha, Jonathan A. Coleman, Alvin C. Goh, Robert C. Smith, Behfar Ehdaie, Andrew J. Vickers, Peter T. Scardino, James A. Eastham, Vincent P. Laudone, Timothy F. Donahue

**Affiliations:** 1 Urology Service, Department of Surgery, Memorial Sloan Kettering Cancer Center, New York, New York; 2 Department of Epidemiology and Biostatistics, Memorial Sloan Kettering Cancer Center, New York, New York; 3 Department of Urology, Naval Medical Center Portsmouth, Portsmouth, Virginia; 4 Urology Service, Department of Surgery, Hendrick Medical Center, Abilene, Texas; 5 Urology Department, Austin Health, Heidelberg, Victoria, Australia; 6 Surgery Department, University of Alberta, Edmonton, Alberta, California; 7 Instituto Oncológico, Fundación Arturo Lopez Perez, Santiago, Chile; 8 Office of Technology Development, Memorial Sloan Kettering Cancer Center, New York, New York; 9 Clinical Grade Production Core Facility, Memorial Sloan Kettering Cancer Center, New York, New York; 10 Organic Synthesis Core Facility, Memorial Sloan Kettering Cancer Center, New York, New York; 11 Research and Technology Management, Memorial Sloan Kettering Cancer Center, New York, New York; 12 Department of Neurology, Memorial Sloan Kettering Cancer Center, New York, New York; 13 Department of Neurology, Hospital for Special Surgery, Department, New York, New York

## Abstract

**Importance:**

Fluorescence-guided surgery aims to improve intraoperative identification of vital structures. Rizedisben is a myelin-binding fluorophore that fluoresces in the blue light (370-425 nm) spectrum to improve intraoperative nerve identification.

**Objective:**

To determine the optimal safe and clinically effective dose of rizedisben for sustained intraoperative fluorescence of nerve structures.

**Design, Setting, and Participants:**

A single-arm, open-label, phase 1 study was conducted in patients undergoing robot-assisted laparoscopic radical prostatectomy (RALP) at an urban academic cancer center in New York City between January 2023 and October 2024. Using a dose escalation design, increasing doses of rizedisben were administered after safety was assessed at each level until a clinically effective dose was determined. The obturator nerve served as the reference nerve for measuring fluorescence intensity. Eligible patients were 18 years old and older, diagnosed with prostate cancer, and scheduled for RALP. Patients were recruited in preoperative clinic visits once deemed eligible for the study. Those with prior pelvic surgery or radiation, known central or peripheral nervous system disease, current use of neurotoxic medications, recent exposure to phototoxic drugs, or serious kidney or liver dysfunction were excluded.

**Interventions:**

Rizedisben was intravenously administered intraoperatively 30 minutes prior to visualization of the obturator nerve.

**Main Outcomes and Measures:**

Safety was assessed through 45 postoperative days. Fluorescence was measured via subjective intraoperative scoring and by post hoc objective image analysis. Clinically effective dose was defined as achieving sustained fluorescence of the obturator nerve in 3 or more of 5 patients in 2 consecutive cohorts, provided fewer than 20% of patients experienced grade 2 or greater toxicity. Sustained fluorescence was defined as moderate or better fluorescence for 90 minutes or longer. At the clinically effective dose, fluorescence assessments of the neurovascular bundles were included.

**Results:**

Thirty-eight patients (median [IQR] age, 61.5 [57.8-66.3] years) enrolled in and completed the trial. Dosing was escalated from 0.25 to 3.0 mg/kg. There was 1 grade 2 adverse event (rash) possibly attributable to rizedisben. Sustained fluorescence of the obturator nerve was achieved in all patients at 3.0 mg/kg. Prostate neurovascular bundles demonstrated evidence of fluorescence in 8 of 9 (89%) patients at 3.0 mg/kg.

**Conclusions and Relevance:**

In this phase 1 trial of rizedisben, the 3.0-mg/kg dose was shown to be generally well tolerated and clinically effective. At this dose, there was excellent sustained fluorescence of the obturator nerves, and the neurovascular bundles were visualized in 8 of 9 patients. Based on these data, we are designing phase 2 studies with rizedisben for additional indications.

**Trial Registration:**

ClinicalTrials.gov Identifier: NCT04983862

## Key Points

**Question:**

What is the optimal safe and clinically effective dose of rizedisben, a novel myelin-binding fluorophore, to illuminate nerve structures during robotic radical prostatectomy?

**Findings:**

In this nonrandomized clinical trial among 38 patients undergoing robotic radical prostatectomy, rizedisben demonstrated an excellent safety profile at each dose level. Sustained fluorescence of the obturator nerve was observed in every patient at a dose of 3.0 mg/kg with a 15-minute time to onset of strong fluorescence and duration of more than 3.5 hours.

**Meaning:**

Rizedisben was well tolerated and clinically effective in enhancing nerve visualization during surgery in this study.

## Teaser

This nonrandomized clinical trial evaluates the use of rizedisben, a novel myelin-binding fluorophore, in minimally invasive surgery.

## Introduction

Iatrogenic nerve injury is a leading cause of morbidity associated with many surgical procedures, including prostatectomy, herniorrhaphy, thyroidectomy, mastectomy, and complex pelvic surgery.^1,2^ Visualizing and sparing nerves during surgery is critical to avoiding chronic morbidity, pain, and loss of function.^3,4^ Causes for nerve injury include anatomic variability, poor visibility relative to surrounding tissue, and proximity to vital structures. Currently, nerve-sparing techniques rely on anatomical landmark identification or use of intraoperative monitoring devices to verify nerve location based on stimulation of innervated muscles or organs.^5,6^

Fluorescence-guided surgery (FGS) seeks to achieve high-contrast, high-sensitivity visualization of selected anatomy. The fluorescent agent (fluorophore) emits light when excited by a precise wavelength, which can be in both visible and nonvisible spectra.^7,8^ The fluorophore is engineered to bind specific tissue, thereby illuminating targets for excision (eg, tumor cells) or preservation (eg, nerves).^9,10,11^

There are numerous potential applications for intraoperative nerve fluorescence, one of which is radical prostatectomy. This common major urologic surgery has 2 features that make it an ideal model for FGS. First, most of these surgeries are performed using a laparoscopic technique, eliminating ambient light during fluorophore emission. Second, careful nerve dissection is fundamental to its success.^12,13^ The obturator nerves are routinely encountered during pelvic lymph node dissection, and the neurovascular bundles are intimately associated with the prostate periphery, where most prostate cancer forms.^13,14^ Even in experienced hands, the identification of the nerve-sparing plane can be challenging; surgeons must balance oncologic control with preservation of this functional nerve plexus. The delicate neurovascular bundles may be injured during surgery, which can result in urinary incontinence, erectile dysfunction, and impaired long-term quality of life.^3,4^ Similarly, damage to the obturator nerve can result in marked gait abnormalities. The potential value of intraoperative nerve identification via FGS is evident.

Rizedisben (Illuminare-1; Illuminare Biotechnologies) is a novel small-molecule fluorophore that binds to myelin and fluoresces in the blue light (370-425 nm) spectrum to enhance nerve visualization.^15^ We conducted a clinical phase 1 nonrandomized clinical trial of rizedisben during robot-assisted laparoscopic radical prostatectomy (RALP) to assess the safety of the agent and to identify a clinically effective dose at which sustained visualization of the nerves was achieved.

## Methods

### Trial Structure

This was a single-arm, open-label, phase 1 study of rizedisben use in patients undergoing RALP. The study was carried out in compliance with the Memorial Sloan Kettering Cancer Center Institutional Review Board–approved protocol and the principles of good clinical practice, as described by standard operating procedures, US 21 Code of Federal Regulations dealing with clinical studies (including parts 50 and 56 concerning informed consent and institutional review board regulations) and the Declaration of Helsinki and amendments concerning medical research in humans. Written informed consent was obtained from all patients before enrollment. The trial protocol is provided in Supplement 1.

Patients were recruited in preoperative clinic visits once deemed eligible for the study. Eligible patients were at least 18 years old with a diagnosis of localized prostate cancer scheduled for RALP. Patients with prior pelvic surgery or radiation, known central or peripheral nervous system disease, current use of neurotoxic medications, recent exposure to phototoxic drugs (discontinuation less than 5 half-lives), or serious kidney (creatinine clearance less than 60 mL/min) or liver dysfunction (liver function tests more than 2 times the institutional upper limit) were excluded. Prostate cancer management was not altered based on trial enrollment.

The phase 1 trial consisted of 2 parts. Part 1 was structured as a dose escalation phase to identify the optimal safe and clinically effective dose for sustained fluorescence of nerve structures. The obturator nerve was selected as the reference nerve for fluorescence evaluation in part 1 due to its sufficient myelination and routine exposure during pelvic lymph node dissection. Part 2 included an expansion cohort at the dose found to be well tolerated and sufficient for sustained fluorescence, with the addition of fluorescence assessments of the neurovascular bundles. Safety assessments were performed throughout the entire trial.

### Fluorescence Evaluation

Rizedisben was injected intravenously approximately 30 minutes prior to anticipated visualization of the obturator nerve; this essentially coincided with the beginning of the surgery during docking of the robot. Nerve visualization was performed with D-Light C photodynamic diagnostic rigid system (Karl Storz SE & Co) with a minor modification to a standard 10-mm 0° lens laparoscope, under white and blue light conditions. A snap-on adapter with blue light filter was placed between the camera and the laparoscope lens. Fluorescence was measured subjectively by a study physician via a Likert scale (1-5 points) intraoperatively at approximately 30-minute intervals (1 = no fluorescence of reference nerve; 2 = no difference in contrast between background enhancement and reference nerve; 3 = minimal contrast between reference nerve fluorescence and background normal tissues; 4 = moderate contrast between reference nerve fluorescence and background normal tissues; 5 = maximal contrast between reference nerve fluorescence and background normal tissues). Sustained fluorescence was defined as a subjective score of 4 or more points (moderate or better contrast between reference nerve fluorescence and background normal tissues) for 90 minutes or longer. Postoperatively, objective image analysis was performed by a contract research services company (Visikol) via ImageJ software on operative images of the obturator nerve at time points corresponding to intraoperative assessments.^16^ Pixel intensity analysis was performed on regions of interest of nerve and background fat tissue for comparative measures.

### Dose Escalation

**Figure 1.**
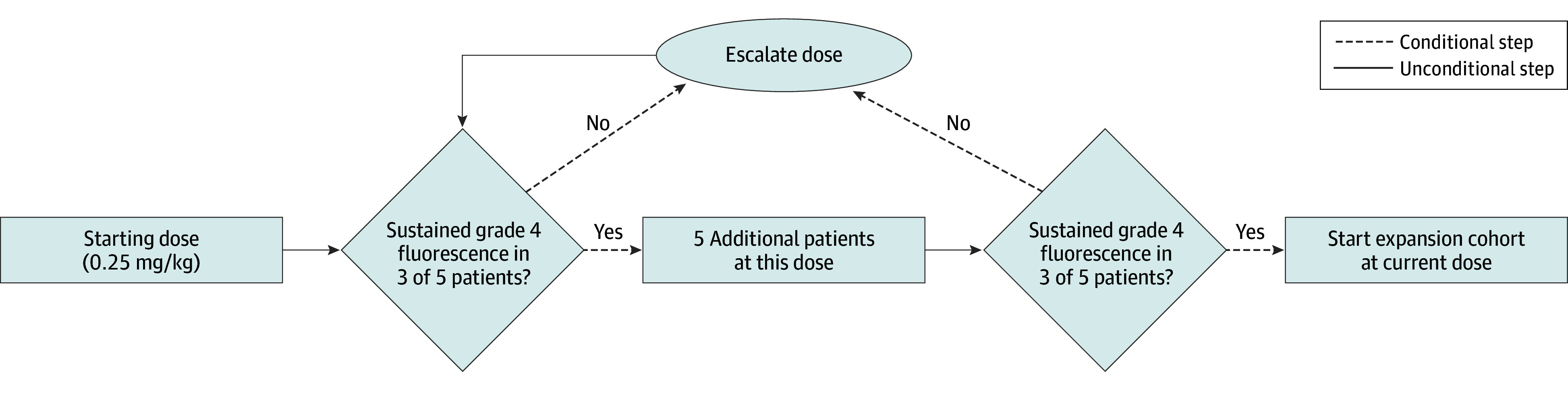
Dose Escalation Study Schema Sustained fluorescence of the obturator nerve was defined as a subjective score of 4 or more points (moderate or better contrast between reference nerve fluorescence and background normal tissues) for 90 minutes or longer. If more than 20% of patients within a cohort experienced a grade 2 or higher adverse event that was considered attributable to the drug and clinically significant, dose escalation was to be stopped at that level, and the prior dose was to be considered the maximum tolerated dose. Adverse events at the first postoperative visit for catheter removal (day 10, ±5 days) were used to determine whether dose escalation could proceed during this phase of the study.

Dosing was initiated at 0.25 mg/kg based on preclinical animal data.^15^ At each dose level, if 3 or more of 5 patients achieved sustained fluorescence, a second 5-patient cohort was added at that dose. If sustained fluorescence was not achieved in 3 or more of 5 patients, the dose was escalated by no more than 0.75 mg/kg. Clinically effective dose was defined as achieving sustained fluorescence in 3 or more of 5 patients in 2 consecutive cohorts, provided fewer than 20% of patients experienced Clavien-Dindo grade 2 or greater toxicity (Figure 1). Dose escalation proceeded until a sustained fluorescence was achieved, up to a maximum dose of 3.0 mg/kg.

### Safety Evaluation

In addition to routine presurgical evaluation, enrolled patients underwent neurologic evaluation using the Neuropathy Impairment Scale–Lower Limbs (NISLL) and Beijers questionnaire for Common Toxicity Criteria Grading of Peripheral Neuropathy at baseline (preoperatively on day of surgery), 24 hours postoperatively, day 10 (±5 days), and postoperative day 45 (±10 days).^17,18^

Serious adverse events (AEs) were reported from the time of informed consent to the end of study evaluation (mean [SD] postoperative day 45 [10]). To assess the degree of causality attributable to rizedisben exposure, each AE was reviewed by the clinical team then assigned a score of not related, unlikely, possible, probable, or definite. If the causality of an AE was scored as possible or above, the AE was also scored according to Clavien-Dindo classification.^19^ Grade 2 or higher AEs were considered clinically significant.

Blood draws for pharmacokinetic evaluation were performed at regular perioperative and postoperative intervals (data to be reported separately). Electrocardiograms were performed at time points corresponding to perioperative pharmacokinetic evaluations via Holter monitor.

### Statistical Analysis

The association between dose and fluorescence scoring was estimated using generalized estimating equations with an exchangeable correlation structure adjusting for both a linear and quadratic term for time from the start of surgery, as we expected the association between time and visualization to be nonmonotonic. Outcomes of interest included an objective measure of visualization and a subjective assessment on a scale of 1 to 5. For the subjective assessment, we dichotomized the outcome as a score of 4 or higher, as this was considered effective identification of nerves sufficient to guide surgery. Accordingly, a logit link was used. We tested for interactions between dose and both the linear and quadratic term for time using a simultaneous Wald test and maintained the interaction terms where significant. Analyses were conducted using R version 4.4.0 (R Foundation) with the geepack version 1.3.12 package.^20^

## Results

Between January 2023 and October 2024, 38 patients (median [IQR] age, 61.5 [57.8-66.3] years) at an urban academic cancer center in New York City were enrolled in and completed the trial. Demographic and dose level data are presented in Table 1. Four patients experienced AEs relevant to rizedisben administration. Three AEs were scored as possibly related to rizedisben: peripheral sensory neuropathy, paresthesia, and maculopapular rash. Only the maculopapular rash was a Clavien-Dindo grade 2 AE (the rest were grade 1) in a patient with considerable dermatologic history. One AE—grade 1 photophobia—was scored as definitely related to rizedisben and resolved without intervention in less than 48 hours. In total, there were 31 grade 2 AEs among 20 patients and 2 grade 3 AEs among 2 patients; there were no grade 4 or 5 AEs. Apart from the AEs described above, the rest were all scored as not related or unlikely to be related to rizedisben (Table 2). There were no significant cardiac events detected on Holter monitor, and echocardiograms on postoperative day 1 demonstrated no new significant findings.

**Table 1.** Demographic and Clinical Data by Dose Level

Variable	Dose level						
	0.25 mg/kg (n = 3)	0.50 mg/kg (n = 3)	1.0 mg/kg (n = 10)	1.5 mg/kg (n = 5)	2.0 mg/kg (n = 4)	2.25 mg/kg (n = 4)	3.0 mg/kg (n = 9)
Age, median (IQR), y	61 (61-77)	60 (59-62)	62 (57-66)	60 (50-65)	61 (55-66)	58 (53-64)	66 (61-68)
Body mass index, median (IQR)^a^	28 (25-29)	32 (29-40)	32 (27-36)	30 (27-35)	29 (27-34)	27 (24-27)	27 (26-29)
Dose, median (IQR), mg	21 (18-24)	50 (47-60)	102 (84-124)	145 (128-170)	188 (170-220)	177 (154-198)	252 (248-278)
Attributable adverse events, No.^b^							
Grade 1	0	0	0	0	1	0	2
Grade 2	0	0	1	0	0	0	0
Any moderate fluorescence, frequency, %^c^	0	0	8 (80)	5 (100)	3 (75)	4 (100)	9 (100)
Sustained fluorescence, frequency, %^d^	0	0	4 (40)	2 (40)	3 (75)	3 (75)	9 (100)

^a^ Calculated as weight in kilograms divided by height in meters squared.

^b^ Attributable adverse events were assigned a designation by the likelihood of the adverse event being caused by exposure to the study drug. The nature of the event, temporal sequence relative to drug administration, and potential alternative causes were considered. Adverse events categorized as possibly, probably, or definitely related to rizedisben administration were scored according to Clavien-Dindo classification.

^c^ Based on observations of the obturator nerve, defined as ≥4 points.

^d^ Sustained fluorescence was defined as a subjective score of ≥4 points (moderate or better contrast between reference nerve fluorescence and background normal tissues) for ≥90 minutes.

**Table 2.** Summary of Adverse Events^a^

Adverse event grade	Degree of causality attributable to rizedisben, No.					Total
	Not related	Unlikely	Possible	Probable	Definite	
1	98	9	2	0	1	110
2	24	6	1	0	0	31
3	1	1	0	0	0	2
Total	123	16	3	0	1	143

^a^ Attributable adverse events were assigned a designation by the likelihood of the adverse event being caused by exposure to the study drug. The nature of the event, temporal sequence relative to drug administration, and potential alternative causes were considered. Adverse events categorized as possibly, probably, or definitely related to rizedisben administration were scored according to Clavien-Dindo classification. There was 1 grade 2 adverse event (maculopapular rash) deemed possibly related to rizedisben administration. There were no grade 4 or 5 adverse events experienced in the study population.

Beijers questionnaires were completed by 37 of 38 patients (97%) on postoperative day 1, 36 patients (95%) on postoperative day 10, and 30 patients (79%) on postoperative day 45. At postoperative day 45, 12 of 30 (40%) reported at least 1 symptom that had worsened compared to their answer at baseline. The most frequently reported symptoms were problems achieving erection (n = 8) and frequent urination (n = 4), which are expected findings in the postprostatectomy setting. Two patients reported any change in sensation, but only 1 specified the symptom as numbness in the fingers. Postoperative NISLL evaluations were completed for all patients. Four patients (11%) were found to have any lower extremity neurologic changes on postoperative day 1, but only 2 patients (5%) had any NISLL findings different from baseline at postoperative day 10. Symptoms included mild triceps surae weakness bilaterally in 1 patient and decreased pinprick sensation bilaterally in the second patient. By postoperative day 45, no patients had any deficits on the NISLL compared to baseline.

**Figure 2.**
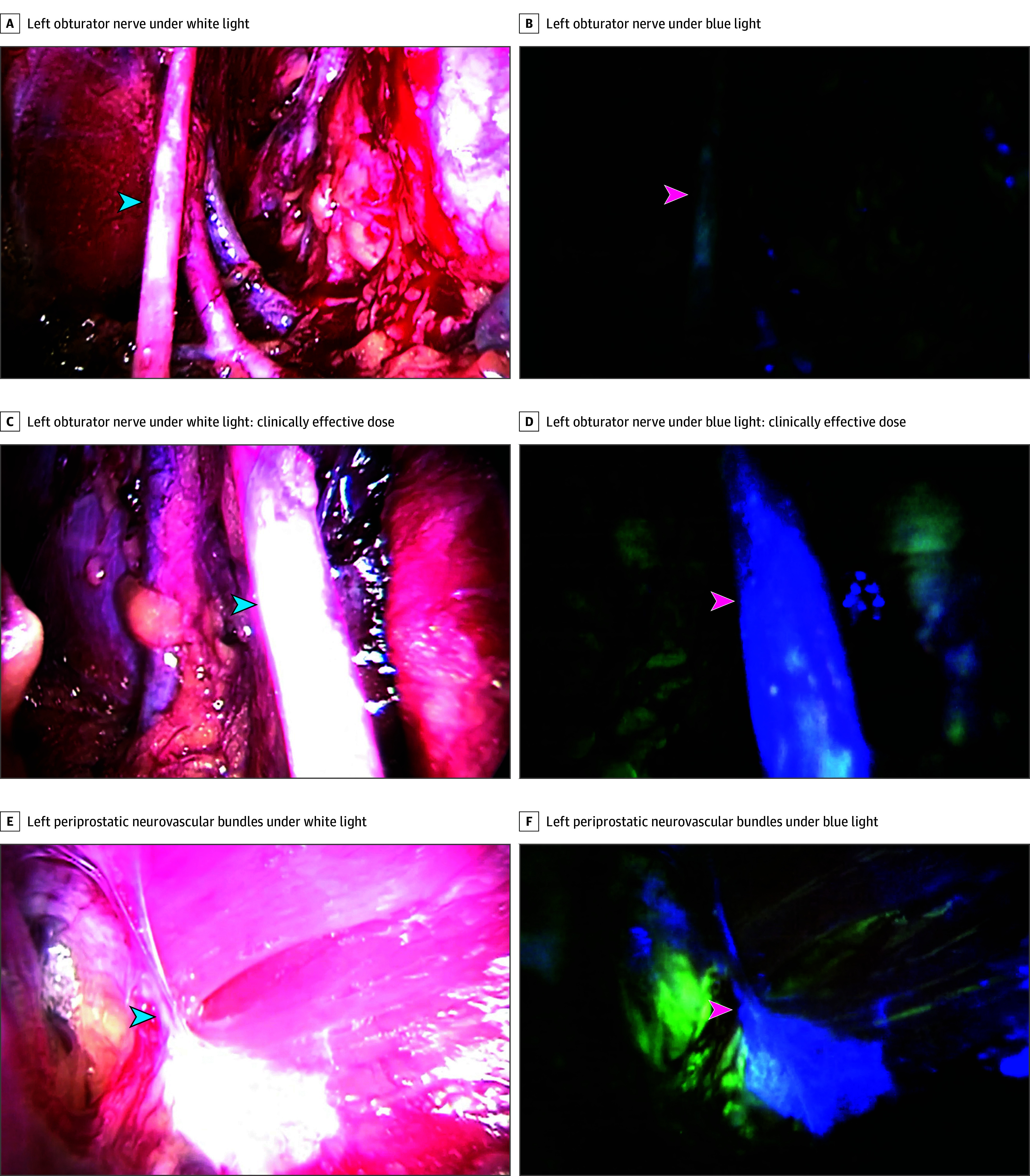
Comparison of Target Nerve Visualization Under White Light and Blue Light Conditions A and B, Imaging of the left obturator nerve (indicated by arrowheads) was obtained at 85 minutes postinjection with a rizedisben dose of 1.0 mg/kg. C and D, Imaging of the left obturator nerve (arrowheads) obtained at 75 minutes postinjection with a rizedisben dose of 3.0 mg/kg. E and F, Imaging of the left periprostatic neurovascular bundles (arrowheads) obtained at 140 minutes postinjection with a rizedisben dose of 3.0 mg/kg.

**Figure 3.**
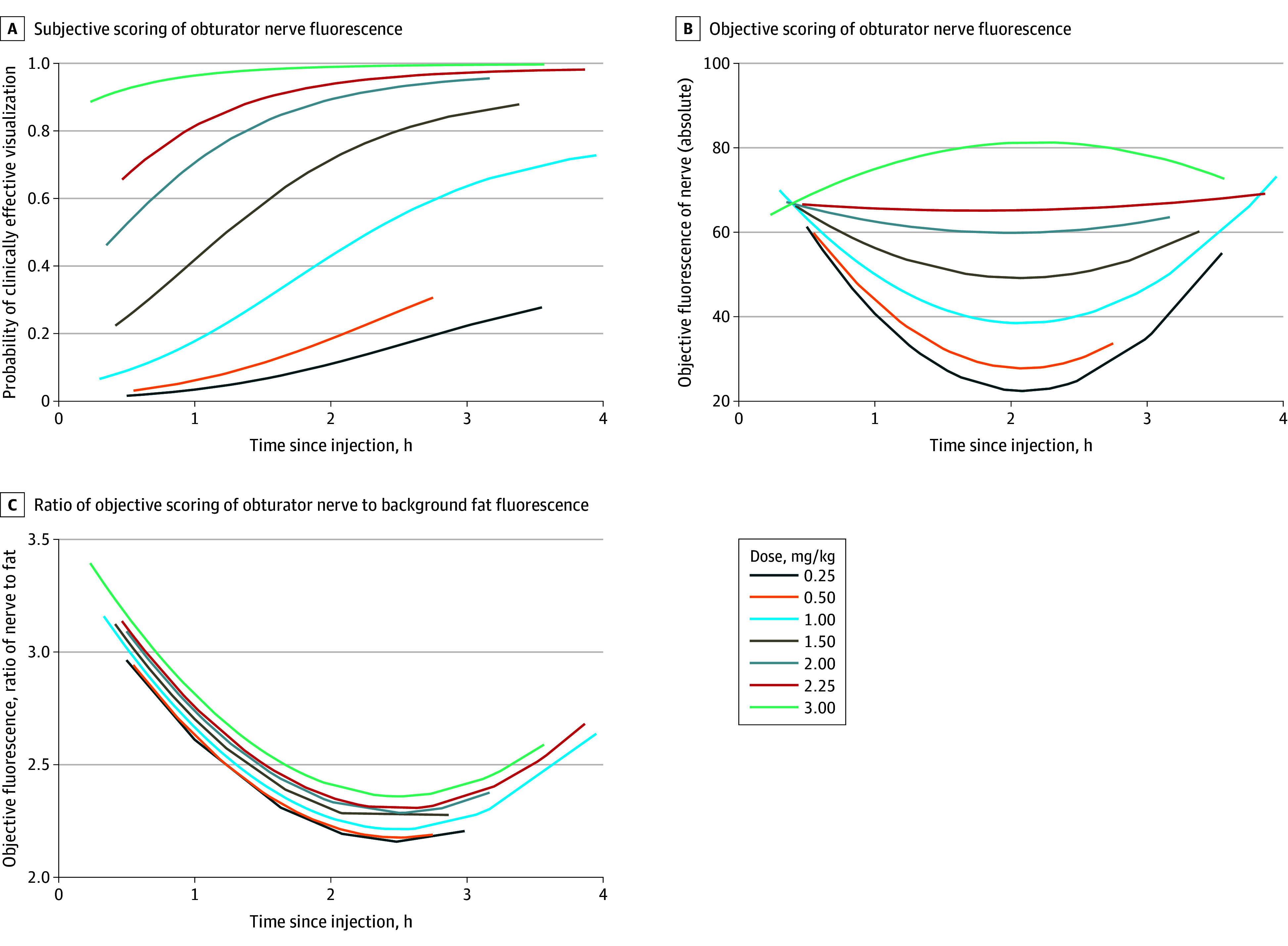
Scoring of Obturator Nerve Fluorescence A, For subjective scoring, clinically effective visualization was defined as 4 or 5 of 5 by intraoperative surgeon assessment, estimated using generalized estimating equations. B, Post hoc objective image analysis was performed by Visikol. Fluorescence is presented as the mean intensity of pixel values in regions of interest as measured by ImageJ software, estimated using generalized estimating equations. C, Fluorescence is presented as the mean intensity of pixel values in regions of interest as measured by ImageJ software, estimated using generalized estimating equations.

The proportion of cases in which sustained fluorescence of the obturator nerve was achieved increased with sequential dose escalation. No patients achieved sustained fluorescence until the dose was increased to 1.0 mg/kg, while all patients achieved sustained fluorescence at 3.0 mg/kg (Table 1). With dose escalation, the clinically effective dose was achieved at 3.0 mg/kg. At the clinically effective dose, moderate or better fluorescence (subjective score of 4 or more points) of the obturator nerve was observed consistently (41 of 42 observations), ranging from 14 minutes to more than 3.5 hours postinjection. There was no evidence of diminishing fluorescence at the last observations prior to surgery conclusion, so the true duration of fluorescence is not yet known. Representative intraoperative images are presented in Figure 2. The probability of moderate or better subjective fluorescence was significantly associated with increasing rizedisben dose (odds ratio, 11.1 per mg/kg; 95% CI, 4.3 to 28.8; *P* < .001) and a positive association was seen between the probability of moderate or better subjective fluorescence and duration after injection (Figure 3A). On post hoc image analysis, objective measure of absolute fluorescence of the obturator nerve increased significantly with dose (coefficient, 14 per mg/kg; 95% CI, 6.1 to 22.4; *P* < .001) (Figure 3B). The ratio of objective fluorescence of the obturator nerve compared to fluorescence of background fat was also measured, but there was no evidence that dose was associated with the objective visualization ratio (coefficient, 0.07; 95% CI, −0.32 to 0.47; *P* = .72) (Figure 3C).

At the clinically effective dose, assessment of neurovascular bundle fluorescence was performed at various points during the prostatic dissection. Neurovascular bundles demonstrated no observed fluorescence in 1 patient (11%), score 3 of 5 fluorescence in 5 patients (56%), and score 4 of 5 fluorescence in 3 patients (33%) at 3.0 mg/kg. There was no observed fluorescence of the neurovascular bundles at doses less than 3.0 mg/kg.

## Discussion

We conducted a phase 1 nonrandomized clinical trial using rizedisben, a novel myelin-binding fluorescent small molecule, to perform real-time intraoperative identification of nerve structures as an adjunct to white-light visualization. We evaluated the first in-human use of rizedisben during RALP. The trial was structured as a sequential dose escalation study. The primary objectives were to evaluate the safety of rizedisben and determine a clinically effective dose, which yielded sustained fluorescence of the obturator nerve.

Rizedisben was generally well tolerated at all dose levels. The most significant AE was a grade 2 rash seen in 1 patient; this was deemed possibly related to rizedisben given the patient’s notable dermatologic history. One grade 1 AE of photophobia, lasting less than 48 hours, was deemed definitely related to rizedisben. This AE resolved without intervention and was not associated with other AEs. There were minimal neurologic changes based on the survey and physical examination evaluations, most of which were urinary changes commonly seen during the natural healing process after prostate removal and urinary tract reconstruction.

The clinically effective dose was identified after escalating doses were administered, safety was evaluated at each level, and sustained fluorescence was documented by both subjective intraoperative assessments and objective post hoc imaging analyses. Strong fluorescence was seen with rapid onset (less than 15 minutes) and with durable effect (more than 3.5 hours). From a practical standpoint, this allows for intraoperative flexibility since rizedisben would not need to be infused hours or days in advance of surgery, and duration of fluorescent activity allows for use at any point in most major surgeries. There was no observed fluorescence of the neurovascular bundles at lower doses, which supports the clinical utility of the 3.0 mg/kg dose. In the expansion cohort, neurovascular bundles demonstrated fluorescence with clear contrast between the diminutive nerve tissue and the adjacent prostate capsule. This is critically important since intraoperative identification of the neurovascular bundles (relative to the obturator nerve) is more challenging in routine radical prostatectomy. The intensity of neurovascular bundle fluorescence was less intense than that of the obturator nerve, likely due to less robust myelination and smaller size. Nevertheless, these results are encouraging and there is considerable opportunity for use of FGS in nerve dissection, which may play a crucial role in preserving erectile and urinary function.^3,13^

The most commonly used fluorophores with clinical approval for FGS are indocyanine green, methylene blue, 5-aminolevulinic acid/hexaminolevulinate, and fluorescein.^8^ These agents have demonstrated success in angiography, lymphography, cholangiography, and tumor identification.^10,21,22,23,24,25^ However, limitations in tissue specificity, fluorescence intensity, and photostability have created opportunities for more sophisticated fluorophore engineering, leading to the introduction of antibody-conjugated and small molecule probes, such as rizedisben.^8,10,15^ Fluorescent probes targeting nerve tissue have existed for several decades but have been hampered by challenges inherent to nerve biology. As examples, axonal transport mechanisms slow intracellular uptake (challenging their flexibility for surgical use), and lipophilicity of probes causes background fluorescence of adipose tissue (confounding precise visualization).^8,11,15,26^ Results of this phase 1 trial demonstrate that rizedisben overcame both obstacles in vivo. Not only were there obvious visual differences between the signal intensities of the target nerves and background tissue, but the different tissue types demonstrated stark color contrasts, accentuating anatomical contours of the nerve structures. For example, while nerves maintained a blue fluorescence, fat showed a bright green signal under blue light (Figure 2). This is a possible explanation behind the findings of the objective analysis of nerve to fat fluorescence ratios. Adjacent tissue signal intensity may have increased to levels closer to the target nerve signal intensity, but color contrasts (which are not measured objectively) made the strong fluorescent contrast evident to the surgeon.

There are currently no approved intraoperative agents to aid in the visual identification of nerve structures. Typically, surgical procedures are performed under ambient or white light, without image guidance, with reliance on either plain or magnification-assisted visualization for anatomic guidance. It is widely recognized by surgeons that a definitive nerve identification method would be helpful, compared with the currently available option for intraoperative nerve identification, which is nerve monitoring. Nerve monitoring has limitations: (1) it cannot be used on many at-risk nerves; (2) it provides feedback only if stimulation is provided by the surgeon with a probe, which requires nerve identification; (3) it does not work in conjunction with electrocautery; and (4) it can be applied only to motor nerves. The ability to reliably identify nerves (autonomic, motor, sensory) within the operative field would help guide dissection across multiple surgical subspecialties, which may translate into improved surgical and functional outcomes.^1,2,3^

Efficacy of any fluorophore is highly dependent on the hardware and software components that illuminate, capture, and analyze the target tissue. Technical details such as camera orientation and lens distance also influence successful fluorescence visualization.^9,10^ In RALP, the anatomical constraints of the pelvis necessitate positioning of the laparoscopic camera and excitation light source near the target tissue, especially during neurovascular bundle dissection. Light scatter and reflection are more pronounced in enclosed spaces, which in our study increased background noise in some of the expansion cohort patients. Software advances leveraging artificial intelligence are in development to mitigate hardware and technical limitations.

### Limitations

There are limitations of this trial that warrant mention. First, assessments of nerve fluorescence were conducted based on surgeon expertise of the anatomy; pathologic confirmation of nerve tissue was not required as part of the study design. That said, the obturator nerve is a distinct structure separate from surrounding structures and unlikely to be mislabeled. We plan to perform histologic confirmation of small nerves in our phase 2 trials in nerve reconstruction surgeries. Second, subjective fluorescence scoring was susceptible to confirmation bias since the scorers were already familiar with the target anatomy. In addition, the scoring metric was based on relative terms such as *moderate* and *maximal*. As the dose level was escalated, it became evident that perceptions of strength of fluorescence differed (ie, moderate fluorescence at higher dose was relatively stronger than the same scored fluorescence at lower doses). Objective image analysis confirmed that fluorescence did increase with increasing doses, and the overall trends in subjective and objective scoring were consistent, indicating that both methods for fluorescence analysis were effective.

## Conclusions

The results of this trial should motivate additional FGS experiments, including assessments of rizedisben fluorescence in open surgical environments, concurrent use of tumor-specific fluorophores, alternative camera technologies, and live image optimization. The results will be used to design subsequent studies to assess the ability of rizedisben to identify nerve structures, to guide the extent of surgical resection, and to allow preservation of these structures when appropriate.

Rizedisben, a novel small molecule myelin-binding fluorophore, maintained an excellent safety profile at all doses in this phase 1 trial. After dose escalation, the clinically effective dose was reached at 3.0 mg/kg, where sustained fluorescence of the obturator nerve was observed in all patients receiving that dose. The performance of rizedisben evidenced in this phase 1 trial merits further exploration into its surgical utility.

## Notes

Supplement 1. Trial protocol

Supplement 2. Data sharing statement
