# Supplementary material for: Rizedisben in Minimally Invasive Surgery: A Nonrandomized Clinical Trial
Source: JAMA Surg. 2025 Jul 2;160(8):875–83. doi: 10.1001/jamasurg.2025.1987 (PMC12224043; doi:10.1001/jamasurg.2025.1987)
Supplement: Supplement 1. — Trial protocol [file jamasurg-e251987-s001.pdf]

MSK PROTOCOL COVER SHEET

Phase I Trial of Real-Time Intraoperative Fluorescent Imaging of Nerve Structures Using Illuminare-1, A Novel Myelin-Binding Fluorophore

Principal Investigator/Department: Timothy Donahue, MD

Memorial Sloan Kettering Cancer Center
1275 York Avenue
New York, New York 10065

Table of Contents

1.0 PROTOCOL SUMMARY AND/OR SCHEMA 3
2.0 OBJECTIVES AND SCIENTIFIC AIMS 3
3.0 BACKGROUND AND RATIONALE 4
4.0 OVERVIEW OF STUDY DESIGN/INTERVENTION 9
4.1 Design 9
4.2 Intervention 11
5.0 THERAPEUTIC/DIAGNOSTIC AGENTS & NON-THERAPEUTIC ASSESSMENTS 18
6.0 CRITERIA FOR PARTICIPANT ELIGIBILITY 24
6.1 Participant Inclusion Criteria 24
6.2 Participant Exclusion Criteria 25
7.0 RECRUITMENT PLAN 25
7.1 Research Participant Registration 25
7.2 Randomization 26
8.0 INFORMED CONSENT PROCEDURES 27
9.0 PRE-TREATMENT/INTERVENTION 27
10.0 TREATMENT/INTERVENTION PLAN 28
11.0 EVALUATION DURING TREATMENT/INTERVENTION 30
12.0 CRITERIA FOR REMOVAL FROM STUDY 33
13.0 CRITERIA FOR OUTCOME ASSESSMENT AND ENDPOINT EVALUABILITY 33
14.0 BIOSTATISTICS 34
15.0 TOXICITIES/RISKS/SIDE EFFECTS 35
15.1 Serious Adverse Event (SAE) Reporting 37
15.2. External SAE Reporting 40
16.0 PROTECTION OF HUMAN PARTICIPANTS 40
16.1 Privacy 40
16.2 Data Management 41
16.3 Quality Assurance 42
16.4 Data and Safety Monitoring 42
17.0 REFERENCES 44
18.0 APPENDICES 44

1.0 PROTOCOL SUMMARY AND/OR SCHEMA

Title: Phase I Trial of Real-Time Intraoperative Fluorescent Imaging of Nerve Structures Using Illuminare-1, A Novel Myelin-Binding Fluorophore

This is a single-arm, open-label, phase I study of Illuminare-1, a novel myelin-binding fluorophore created with the goal of reducing inadvertent iatrogenic injury during surgery by allowing fluorescent identification of nerves. In Part 1, the dose-escalation phase, we will investigate the safety of Illuminare-1 at a minimum of 3 dose levels in patients undergoing minimally invasive radical prostatectomy and bilateral pelvic lymph node dissection. Additionally, the pharmacokinetics of a single injection of Illuminare-1 and the time to acceptable visualization of the obturator nerve, a universally encountered reference nerve, will be determined. Illuminare-1 will be evaluated at a minimum of 3 dose levels or until dose-limiting toxicities (DLTs) are experienced or sustained nerve visualization is observed. In Part 2, to provide additional safety data, collect data on the ability of the agent to visualize the obturator nerve and periprostatic nerves (to inform the design of a phase II study), and provide data for future hardware and software optimization, an expansion cohort of up to 20 patients will receive the dose identified in Part 1. Subjects will receive standard of care surgery, and Illuminare-1 will not be used to make surgical treatment decisions. Up to 50 patients will take part in the study; study enrollment is anticipated to take less than 18 months with roughly two months of follow up after the last patient receives the agent; total time for the protocol is estimated to be 20 months.

2.0 OBJECTIVES AND SCIENTIFIC AIMS

Hypothesis: Intravenous administration of Illuminare-1 will enhance real-time intraoperative visualization and delineation of nerve structures in a surgical field.

Part 1 – Dose Escalation

Primary Objectives:

- To determine the safety of Illuminare-1 after intravenous administration, as measured by clinical evaluation (24 h post-injection, postoperative day 10 ± 5 days, postoperative day 30 ± 5 days, and postoperative day 45 ± 10 days).
- To determine the dose that achieves sustained acceptable fluorescence of the obturator nerve using a subjective intensity scale assessed during surgery.

The dose with acceptable nerve to muscle ratio (NMR) fluorescence that does not result in DLTs will be chosen as the ready-for-phase-II dose and will be the dose for Part 2 of the study.

Secondary Objective:

- To assess the pharmacokinetics of Illuminare-1 at each dose level tested.

Part 2 – Expansion Cohort

Primary Objective:

- To obtain clinical safety assessments of Illuminare-1, after intravenous administration, at specified time points (24 h post-injection, postoperative day 10 ± 5 days, postoperative day 30 ± 5 days and postoperative day 45 ± 10 days).

Secondary Objectives:

- To continue visual assessment of the obturator nerve using a subjective intensity scale assessed during the course of surgery.

Exploratory Objectives:

- For patients in the expansion cohort once the safe and effective dose has been identified, to assess visualization of the neurovascular bundles under blue-light fluorescence, compared with white-light, at multiple time points during standard of care minimally invasive radical prostatectomy.
- To correlate the *in vivo* and/or *ex vivo* fluorescent signal of any presumed nerve tissue with corresponding histologic evaluation in select patients undergoing a wider surgical resection as part of routine surgery (i.e., patients with higher-stage disease for whom non-nerve-sparing approaches would normally be used). This would apply to all patients in the study where applicable.

3.0 BACKGROUND AND RATIONALE

a) Unmet Clinical Need

Iatrogenic nerve injury is a leading cause of morbidity associated with many common surgical procedures, including prostatectomy, herniorrhaphy, thyroidectomy, mastectomy, and complex pelvic surgery, among others. Circumstances resulting in nerve injury during laparoscopic and open surgical procedures are highly variable and include anatomic variability, poor visibility of nerves relative to surrounding tissue, and proximity of the nerve to vital structures that may be encountered or resected as part of surgery. Complications arising from these injuries may result in loss of function or sensation, muscle atrophy, and chronic neuropathy.

Currently, nerve-sparing techniques rely primarily on anatomical landmark identification or the use of intraoperative monitoring devices to verify nerve location on the basis of stimulation of innervated muscles or organs.

There are currently no approved intraoperative agents to aid in the visual identification of nerve structures. Typically, surgical procedures are performed under ambient or white light, without

image guidance, with reliance on either plain or magnification-assisted visualization for anatomic guidance. It is widely recognized by surgeons that a definitive nerve identification method would be helpful, compared with the currently available option for intraoperative nerve identification, which is nerve monitoring. Nerve monitoring has the following limitations: 1) it cannot be used on many at-risk nerves; 2) it provides feedback only if stimulation is provided by the surgeon with a probe, which requires recognizing the nerve; 3) it does not work in conjunction with electrocautery; and 4) it can be applied only to motor nerves. The ability to reliably identify nerves (autonomic, motor, sensory) within the operative field, especially those too small to discern easily, would help guide dissection across multiple surgical subspecialties, which may translate into improved surgical and functional outcomes.[1-3]

We propose to conduct a phase I trial using a novel fluorescent, myelin-binding agent to perform real-time intraoperative identification of nerve structures as an adjunct to white-light (naked eye) visualization. The results will be used to design subsequent studies to assess the ability of Illuminare-1 to identify nerve structures, compared with the use of white light; to improve surgical outcomes by providing real-time identification of nerve structures; to guide the extent of surgical resection; and to allow preservation of these structures when appropriate.

b) Nonclinical Studies

A series of nonclinical studies were conducted to demonstrate proof-of-concept imaging of nerves *in vivo* and to evaluate the safety profile of a single intravenous injection of Illuminare-1. *In vivo* pharmacology studies established that the mechanism of action of Illuminare-1 involves specific binding to myelin basic protein and fluorescence of the molecule upon excitation with blue light (range, 370 to 425 nm). Preliminary, proof-of concept imaging and safety data on the mouse, rat, and pig have been published.[4]

In *in vivo* efficacy studies in pigs and dogs that investigated the use of the clinical device that is intended for use in this protocol (Karl Storz Photodynamic Diagnostic device), fluorescent NMR was 1.45 to 1.55, between 30 and 120 min, as measured by objective analysis (at dose levels between 0.25 and 1 mg/kg in pigs). The maximum fluorescent signal was observed at approximately 30 min after injection in both species and was sustained for >2 h. The effective dose was 1.0 mg/kg.

Pharmacokinetics studies in rats and dogs showed similar terminal half-lives, between 4 and 6 h, for both males and females. Metabolism studies revealed that metabolism is comparable in human and dog hepatocytes (with high metabolic stability), whereas in minipig and rat hepatocytes there was a slightly lower percentage of parent remaining.

The toxicology profile of intravenously administered Illuminare-1 has been established in safety pharmacology studies, single-dose intravenous toxicity studies in Sprague-Dawley rats and beagle dogs, *in vitro* and *in vivo* genetic toxicity studies, a local tolerance study, and an *in vitro* phototoxicity study.

After a single intravenous (slow bolus) administration of Illuminare-1 in rats, the principal toxicities were seen only at dose levels ≥30 mg/kg. Findings on Day 2 included histopathologic changes in the epididymis (degeneration/necrosis, sperm stasis, and mixed inflammation), kidney (hyaline casts, tubular degeneration, mixed cell infiltrate, and fibrin thrombi), and administration site (hemorrhage, necrosis, neutrophilic inflammation, and edema). Histopathologic changes were still present at the end of the 14-day recovery period in the kidney (hyaline casts, glomerulonephritis, fibrosis, tubular degeneration/regeneration, and mononuclear inflammation), epididymis (sperm granuloma and mononuclear inflammation), and administration site (fibrosis, tunica intima fibroplasia, mononuclear inflammation, and pigment). In the kidneys, there was no recovery of hyaline casts and partial recovery of tubular degeneration at 14 days. Additionally, a new finding of glomerulonephritis was diagnosed. In the epididymis, there was complete recovery of epithelial degeneration/necrosis and sperm stasis, but there was a new finding of sperm granulomas, which was interpreted as likely representing the natural progression of previous areas of epididymal degeneration/necrosis and sperm stasis. At the administration site, there was partial resolution of the changes. In addition, there were macroscopic observations noted at postmortem examination in the epididymis, kidney, and administration site (tail vein). There were also some organ weight changes in the liver (increased), testis (decreased), adrenal gland (increased), spleen (decreased), and lung (increased), although there were no histopathologic correlates to these effects on organ weights. There were some changes in liver chemistries that could correlate with increased liver weights, including increased AST and ALT.

To further investigate the changes seen through 14 days after administration of the agent, we conducted similar toxicity studies in rats with a longer (30 day) recovery period to assess the degree of persistence or resolution of toxicities. These showed complete recovery of all findings by day 30, except 2 instances of minimal hyaline casts in kidneys (1 male at 20 mg/kg and 1 female at 45 mg/kg); these were considered non-adverse.

Some rats died after single intravenous doses ≥90 mg/kg during experiments designed to push to the highest maximum tolerated doses of the drug. The cause of death was undetermined. The ≥ 90 mg/kg dose is approximately 60-fold higher than the planned starting dose in humans of 0.25 mg/kg, based on body surface area calculations.

In safety pharmacology studies, no adverse findings were observed for any of the major organ systems evaluated.

Illuminare-1 was not mutagenic in a bacterial reverse mutation assay (Ames test) but did cause chromosome aberrations in an *in vitro* human peripheral lymphocyte assay under certain test conditions. When Illuminare-1 was given to rats by intravenous bolus injection as a daily dose for 3 days (dose levels up to 65 mg/kg/day), it was not genotoxic in either the comet assay with the liver or the bone marrow micronucleus assay. When considering the results of all the genotoxicity studies together, the weight of evidence demonstrates that Illuminare-1 is not genotoxic.

In an *in vitro* study in BALB/c 3T3 mouse fibroblasts, Illuminare-1 showed phototoxic potential, with an IC_{50} of 0.650 µg/mL. Additionally, as grade 1 erythema was seen at 24 and 48 hours post-injection on unprotected skin of pigmented rats, precautions will be taken for the first 48 hours after drug administration. Medications with known phototoxic potential will be avoided and patients will avoid excessive ultraviolet light exposure (patients remain in the hospital until the day after surgery per standard of care and will wear opaque clothing once discharged until 48 hours post-injection).

Exposure limits for the first-in-human study were established on the basis of findings in the pivotal single-dose intravenous studies in rats and dogs. The NOAEL in the dog study was 18 mg/kg (the highest dose tested), and the NOAEL in the rat study was 20 mg/kg. Given the findings in rats, this was considered the most sensitive species, and a conservative approach was taken for the selection of the starting human dose (0.25 mg/kg), which is approximately 13-fold less than the NOAEL in rats. In addition, clinical monitoring of renal function will be performed in this study.

Concentrations of the drug are highest immediately after dosing and decrease relatively rapidly through 2 hours after administration, then progressively more slowly through the last quantifiable time point. Based on the proposed single dose administration to humans and the below-quantifiable-limits of Illuminare-1 (20 mg/kg dose) in rat plasma by 168 hours post-dose, it is estimated that Illuminare-1 will be completely cleared from the systemic circulation in humans before Day 7 after dosing. Potential toxicities that are monitorable in the clinic (neurologic, renal) were evident in rats by Day 2 post-dose, therefore, we expect to see any significant signs of dose-limiting toxicities in patients by day 2 after administration. Moreover, the effects in rats were seen at much higher dose levels compared to the proposed starting dose in humans and the effects seen were also completely resolved in necropsy studies done at 30 days after dosing.

Taken together, these data support the safe use of Illuminare-1 in the proposed patient population under the prescribed dose regimen and in accordance with the proposed clinical protocol.

c) Subjective and Objective Assessment of Fluorescent Nerve Intensity

Assessment of a subjective visual scale of nerve fluorescent intensity was performed in 3 Yorkshire pigs weighing between 30 and 35 kg at dosing levels of 0.1 mg/kg, 0.7 mg/kg, and 1.0 mg/kg, respectively. The subjective intensity scale was modified from one used for measurement of fluorescent intensity in patients who received 5-ALA, an agent that fluoresces under similar blue-light conditions intraoperatively during resection of gliomas.[5-7] Fluorescent intensity of the reference nerve was assessed according to the following scale:

1 = no fluorescence of reference nerve
2 = background enhancement exceeds reference nerve fluorescence
3 = minimal contrast between reference nerve fluorescence and background normal tissues
4 = moderate contrast between reference nerve fluorescence and background normal tissues
5 = maximal contrast between reference nerve fluorescence and background normal tissues

The fluorescent intensity of a reference pelvic nerve in the pig was measured at 0, 1, 5, 10, 15, 20, 30, 45, 60, 75, 90, 105, and 120 min under blue-light conditions. The intensity of fluorescence of the nerve relative to background tissues was recorded by a minimum of 4 and a maximum of 6 observers at each time point. The procedures were recorded in real-time for *post hoc* video still capturing to quantify the nerve to muscle ratios (please note the publication[8] refers to this measurement as SNRs) at each corresponding time point. Calculations were performed according to standard equations and principles. The blue-light-channel image data were quantified after the procedure, by averaging 3 maximum pixel intensities along the length of a given fluorescent structure (nerve). Non-nerve background signal readings were made in triplicate approximately 1-2 cm from the selected nerve. The observable fluorescence of the nerve in respect to adjacent tissues such as muscle and fat was defined as the mean fluorescent intensity (in arbitrary units) of 3 regions of interest (ROIs) in the signal (i.e., nerve structure) minus the mean fluorescent intensity of 3 adjacent background ROIs, divided by the mean fluorescent intensity of the 3 background ROIs. Both subjective observer assessments and quantified the observable fluorescence of the nerve in respect to adjacent tissues such as muscle and fat were plotted along the same graph, with strong concordance between values[8] (Figure 1).

Figure 1.

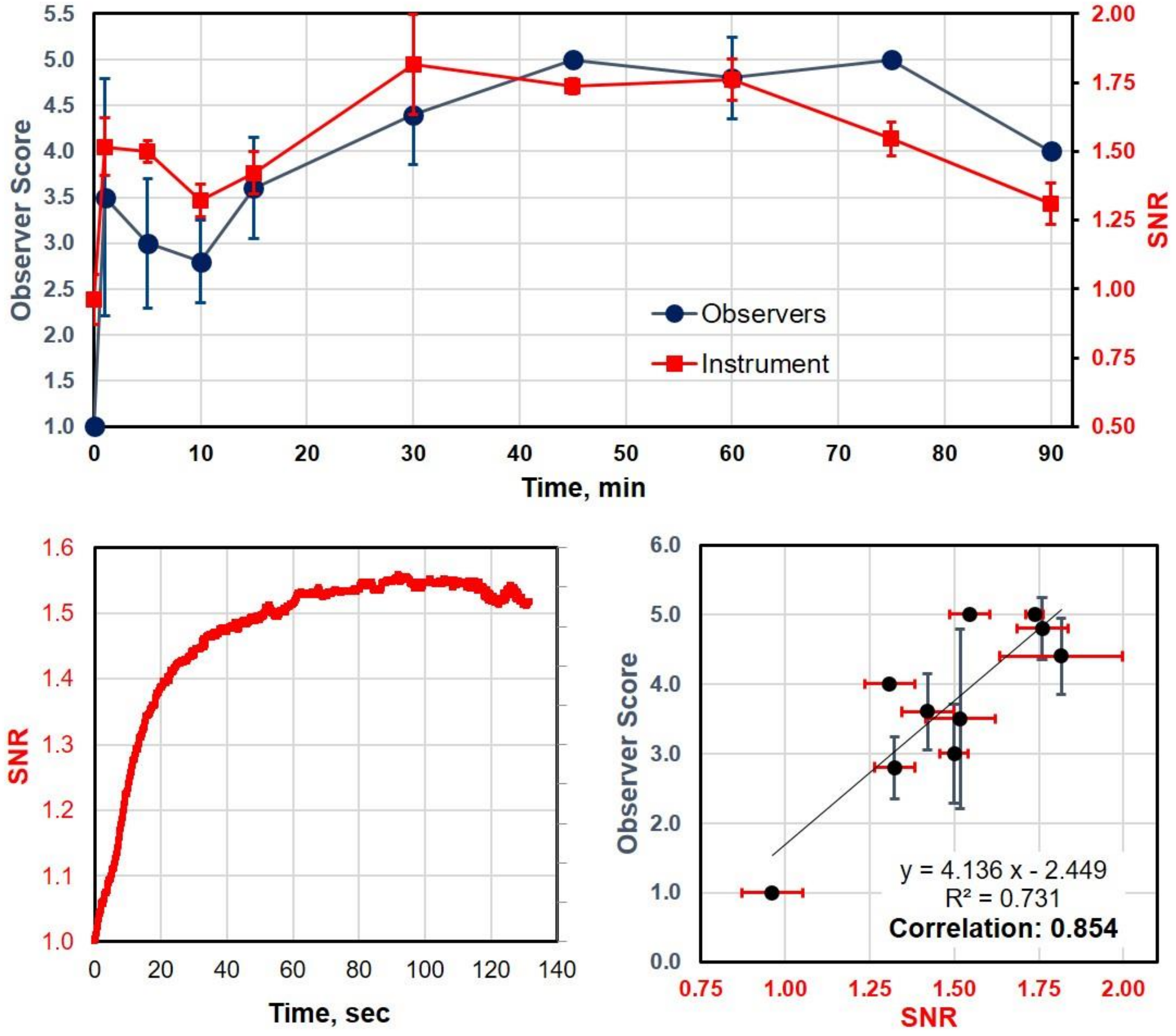

4.0 OVERVIEW OF STUDY DESIGN/INTERVENTION

4.1 Design

This is a first-in-human, single-arm, open-label, dose-escalation phase I study to a) investigate the safety of Illuminare-1, a myelin-binding fluorescent small molecule, in up to 50 patients undergoing routine minimally invasive radical prostatectomy, and b) to investigate the imaging parameters (time to and duration of NMR) for intraoperative obturator nerve visualization and delineation. The degree of fluorescent intensity of the obturator nerve, which will be the reference nerve for the study, will be graded on a 5-point scale scored by a surgeon observer at numerous time points throughout the case, which is based upon prior studies using 5-ALA, an imaging agent that fluoresces in a similar blue-light spectrum, in surgical management of meningiomas.[5-7] Adverse events will be evaluated as described in Section 15. If an adverse

event after administration of the agent occurs, the clinical signs, symptoms, laboratory assessments, and other observations will be recorded. The adverse event will be assessed by the primary investigator to determine if the adverse event was caused by administration of the agent (see section 15 for definitions of causality). The intensity of an adverse event will be graded according to the NCI Common Toxicity Criteria v 5.0. Adverse events that cannot be graded using the NCI Common Toxicity Criteria will be graded as mild (asymptomatic), moderate (symptomatic but not interfering significantly with function) or severe (causing significant interference with function). The pharmacokinetics of Illuminare-1 will be evaluated. Dose levels of the agent will be administered starting at 0.25mg/kg, with increases in dose between cohorts of no more than 0.5 mg/kg until a maximum dose of 2.25mg/kg is reached, if needed. At the dose identified to have the acceptable NMR and no associated DLTs, Illuminare-1 will be studied in up to 20 additional patients at this dose to optimize the imaging protocol and provide additional safety data. Blood samples will be drawn at days 10 (± 5 days) and 45 (± 10 days) and stored for potential future research investigations under MSK IRB#06-107 or MSK IRB#12-245. Neurologic assessments, using the Neuropathy Impairment Scale – Lower Limbs and Beijers questionnaire for CTC Grading of Peripheral Neuropathy, will be performed at enrollment (baseline assessment), during hospitalization after surgery, and at the routinely scheduled postoperative visits at days 10 (± 5 days) and 45 (± 10 days). A phone follow-up will be made at 30 ± 5 days. Video of the white-light and blue-light assessments of the nerves of interest will be recorded throughout the procedure and provided to the investigators and company sponsor for *post hoc* analysis for future hardware and software optimization. Twenty four hour ECGs will be collected via Holter monitor and stored for *post hoc* analysis.

4.2 Intervention

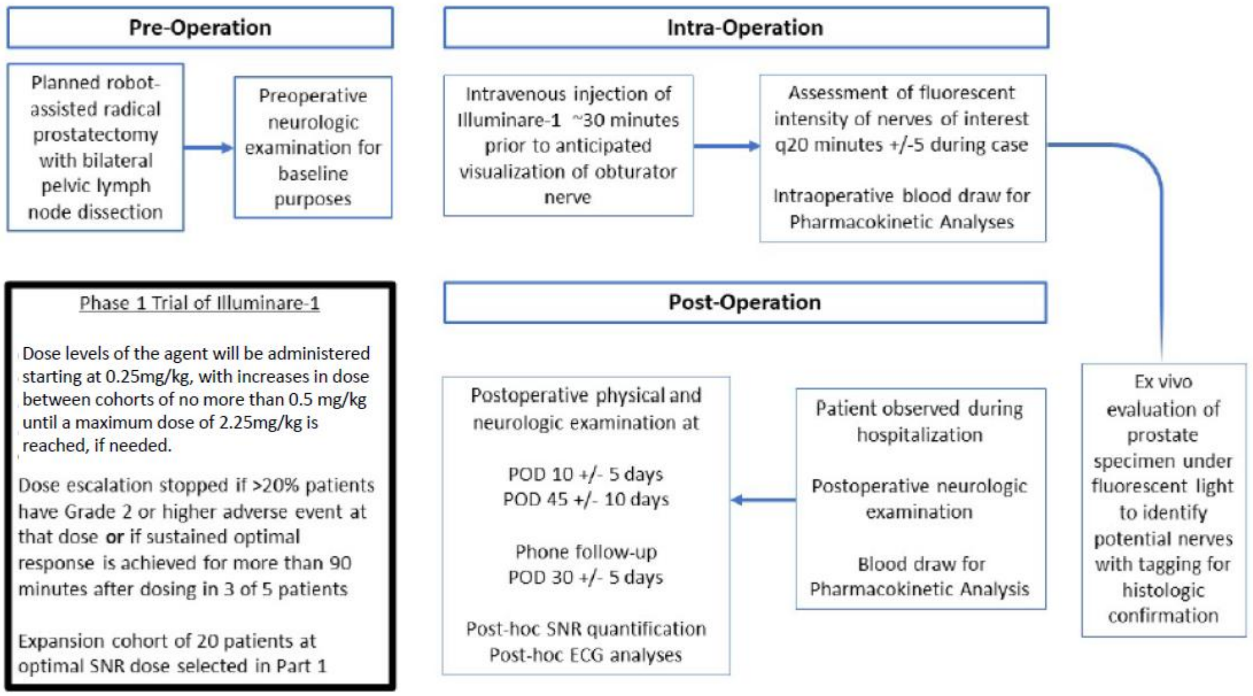

Dose-Escalation Rules:

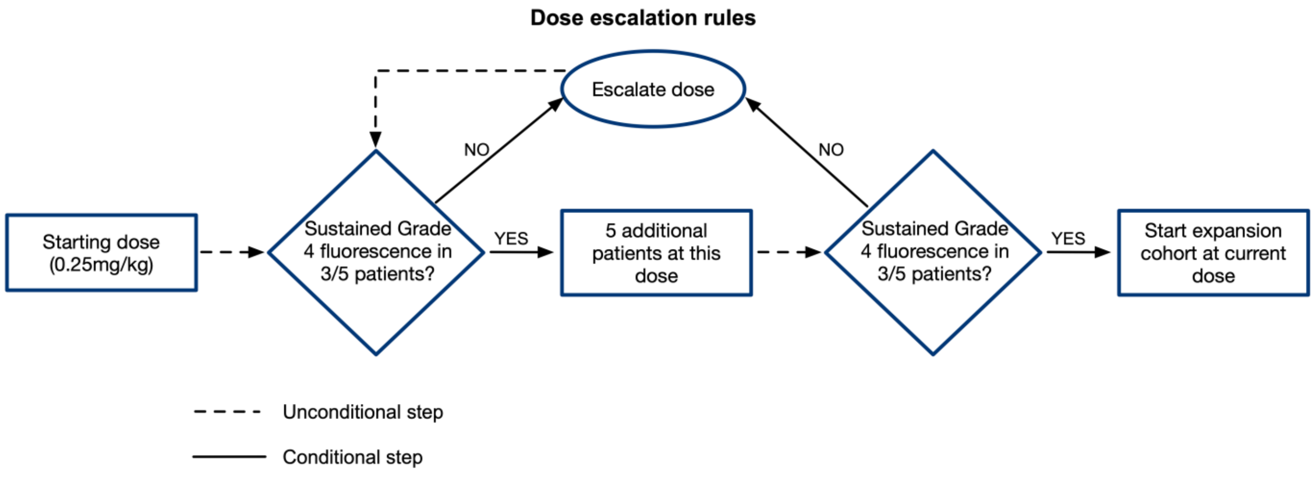

Adverse Event Stopping Rules

If >20% of patients within a cohort experience a grade 2 or higher adverse event that is considered attributable to the drug and clinically significant, dose escalation will be stopped at that level, and the prior dose level will be considered the maximum tolerated dose.

The exact rules are >1 out of 5 for the first five patients, >2 out of 10 in total when an additional five patients added and no more than 20% at any point after that.

Adverse events at the first postoperative visit for catheter removal (10 ± 5 days) will be used to determine whether dose escalation can proceed during Part 1 of the study

In Part 1, 5 patients will initially be observed at each dose level. If >20% of patients within a cohort experience a grade 2 or higher adverse event that is considered attributable to the drug and clinically significant, dose escalation will be stopped at that level, and the prior dose level will be considered the maximum tolerated dose. The exact rules are >1 out of 5 for the first five patients, >2 out of 10 in total when an additional five patients added and no more than 20% at any point after that. Adverse events at the first postoperative visit (10 ± 5 days) will be used to determine whether dose escalation can proceed during Part 1 of the study (see figure above). Based upon short half-life of the agent (approximately 4 hours) and our pre-clinical data, we do not expect to see dose limiting toxicities beyond 2 days after administration of the agent. The first patient receiving the drug will be observed for 30 days before proceeding with administration in other enrolled patients to observe for initial toxicities. We will continue to monitor for both expected and unanticipated toxicities with a phone follow-up at 30 ± 5 day and in-person at the next scheduled clinic visit at 45 ± 10 days. If more than 20% of patients in the first (lowest) cohort have a grade 2 or higher adverse event attributable to the drug, the trial will be stopped.

If sustained subjective grade 4 or higher fluorescence (acceptable fluorescence) is observed for 90 minutes or longer in 3 of 5 patients tested at that dose level, another 5 patients will be enrolled at this dose. If sustained subjective grade 4 fluorescence is not observed in 3 of this second group of 5 patients, this will not be a sufficient dose and escalation to the next highest level will be tested. However, if sustained subjective grade 4 fluorescence is again observed in 3 of the next 5 patients, escalation to an incremental dose (not to exceed +0.5mg/kg) may be assessed in 5 additional patients to measure any potential improvement over the prior dose.

If obturator nerve fluorescence is not sustained at a grade 4 level for at least 90 consecutive minutes in at least 3 of the 5 patients at a dose, the dose will be escalated to the next level, and 5 more patients will be enrolled. This will be repeated sequentially up to the maximum evaluable dose (2.25 mg/kg) if sustained fluorescence is not achieved at a lower dose.

If subjective sustained grade 4 fluorescence is not achieved at any of the dose levels and no DLTs are observed at any dose level, the maximal subjective sustained grade level of fluorescence will be calculated across all dose levels. The lowest dose associated with at least

a subjective sustained grade 3 level of fluorescence will be considered the safe and effective dose.

If sustained grade 3 fluorescence is not achieved in 3 of 5 patients at any dose level, the agent will not be considered to have reached a clinically useful endpoint, and the protocol will not expand directly to Part 2. We will evaluate the safety and clinical data and consider a protocol amendment to the IRB requesting to administer a higher dose than the current maximum in the protocol.

Once the imaging dose of Illuminare-1 is established at a no-DLT level, an expansion cohort of up to 20 patients will be evaluated at this dose. The safety and effectiveness of the agent will be assessed in the same manner as during the dose escalation phase of the study as described above. Briefly, adverse events through the first postoperative visit (10 ± 5 days) will be used to assess for toxicity and if the trial can continue accrual. If >20% of patients experience a grade 2 or higher adverse event that is considered attributable to the drug and clinically significant, this will be considered unacceptable and the trial will be stopped..

Although it is not anticipated to occur, if in the expansion cohort sustained grade 4 fluorescence fails to occur in >25% of patients, this dose will be reevaluated to determine whether it is indeed an acceptable dose for visualization. We will assess the fluorescent response and safety of the next lower and higher doses tested to see whether these need to be explored for further investigation and will submit the appropriate amendment request to the IRB.

In the expansion cohort, observation of the periprostatic nerves in the neurovascular bundle will be included, in addition to assessment of obturator nerve fluorescence as performed in Part 1 of the protocol. One future aim for this agent will be to aid identification of the periprostatic neurovascular bundles, with the goal of improving nerve-sparing surgery. It is not known how well these small neurovascular bundles will fluoresce with this agent in humans and at what time points during surgery the nerves will be visualized. Additionally, with a heavily myelinated nerve such as the obturator, it is unknown whether an optimal signal will result in a similar fluorescent signal in the smaller and variably myelinated nerves in the periprostatic neurovascular bundles. In dog models (which closely resemble human anatomy in the prostatic region), we were able to accurately identify and dissect individual periprostatic nerves as small as 65 microns in diameter; therefore, we anticipate this agent will be able to enhance visualization of similar structures in humans. We will evaluate the neurovascular bundles before opening the endopelvic fascia, during the nerve-sparing portion of the prostatectomy (if performed as part of the standard of care surgery), and once the prostate has been removed.

One surgeon observer will assess whether structures corresponding to presumed nerve tissues within the periprostatic neurovascular bundles can be seen under blue-light conditions (binary result: Yes vs. No). If a positive response is recorded, the surgeon will rate their confidence on a 4-point scale regarding whether the fluorescence represents periprostatic nerves (1 = least confident to 4 = most confident). We will look *ex vivo* at the region of the prostate corresponding to neurovascular bundles after removal of the prostate to see whether

a fluorescent signal consistent with nerve structures within the neurovascular bundle can be identified. Any structures identified *ex vivo* as being consistent with nerve tissue will be tagged for histologic confirmation.

The dose of Illuminare-1 used in the expansion cohort will already have demonstrated relative safety on the basis of the experience from the patients in the dose-escalation part of the study. We will continue to assess adverse events for DLTs in the expansion cohort in the same manner. If >20% of patients experience a grade 2 or higher DLT attributable to the agent, the expansion part of the protocol will be halted, and the trial will be stopped. For example, the agent will be deemed to be unsafe if 5 of 20 patients in the expansion part of the study are found to have significant adverse events attributable to the agent.

A maximum of 50 patients will be enrolled in the trial if each of the first 3 dose cohorts is expanded to 10 patients and 20 patients receive the highest dose level. However, this scenario would mean 3 fluorescent responses in the first 5 patients but not in the second 5 patients, 3 times in succession, which seems unlikely. Our best estimate is that between 30 and 40 patients will be enrolled in the trial.

The human starting dose (0.25 mg/kg) was selected based on the toxicity studies in rats and dogs in which the exposure at the NOAEL in rats (20mg/kg) was approximately 13 times higher than the expected exposure for humans at the starting dose level of 0.25 mg/kg (when adjusted to body surface area). Further consideration was given to investigate an efficacious dose level in humans based on the pharmacology and exposure in mice, rats, dogs and pigs. The time to optimal sustained fluorescence after injection was approximately 30-45 min in dogs and pigs, suggesting an initial time to visualization of 30 min in humans, which will be optimized in this phase I study.

Cognitive and Neurologic Assessment

Despite the absence of any pre-clinical data identifying nerve toxicity, we recognize this agent binds to myelin and there is a need to assess for neurotoxicity. The Beijers questionnaire for peripheral neuropathy uses a series of questions that ask patients about changes in sensation, function, strength, and changes in ability to perform specific activities, such as holding cutlery, walking independently, etc. There is a corresponding key that assigns grade of toxicity based upon the degree of abnormality reported. The Neuropathy Impairment Scale uses a scoring system graduated from 0 points (the normal finding) to a maximum of 88 points (the absence of all motor, sensory, and reflex activity in the lower extremities). The scale is additive of all deficits (64 potential points for muscle strength, 8 points for reflexes, and 16 points for sensory function) in the lower extremities. By combination of the fully validated questionnaires and a brief bedside examination of cognitive and peripheral neural functions, we can closely monitor any possible neurotoxicity which might arise after dose administration. These examinations will be performed at the baseline and according to the schedule listed within the protocol. Any change in these scores will trigger a full neurological investigation within 48 hours.

Patients will receive neurologic assessments at baseline and after drug administration at the following time points: at 24 h after dosing or before discharge from hospital, on day day 10 (± 5 days) post-surgery and at the first postoperative PSA lab check, occurring approximately 7 weeks after surgery (day 45 ± 10 days). Patients will also have a phone follow-up to assess for adverse events at 30 ± 5 days. Patients will undergo baseline assessments of cognitive and neurologic function by use of the following validated instruments: the Beijers questionnaire for CTC grading of peripheral neuropathy (Appendix B) and the Neuropathy Impairment Scale–Lower Limbs (Appendix A). These same validated instruments will be used at the postoperative time points listed above; if a cognitive change from baseline is observed or if a change from baseline scores is identified, a full neurologic assessment will be performed to determine whether the change is considered to be related to the drug. These neurologic assessments are not part of the routine standard of care evaluations performed for patients undergoing minimally invasive radical prostatectomy and are being done as part of the safety profile assessment of the agent.

Patients will be contacted by one of the clinical trials nurses 30 ± 5 days after drug administration for an evaluation since there is no regularly scheduled in-person visit at this timepoint after surgery but we want to continue to assess safety between scheduled visits. We recognize that a physical examination is not possible over the phone, but patients will be assessed for per standard toxicity and grading forms used here at MSK and the assessment can be conducted with a video link between the research nurse and the patient. Telemedicine visits for patients on clinical trials have become more routine during the COVID pandemic as a means of assessing for toxicity and response to therapy. The questions on the two neurologic instruments can be answered either directly by the patient or posed by the nurse to elicit a response. There is one question assessing gait abnormalities visualized when the patient ambulates. We can use video services for this assessment. If is unlikely that difficulties in ambulation will be the only neurologic symptom experienced by patient and it is more likely that the responses to other additional questions would be adequate to determine if there are neurologic changes warranting further evaluation. If any adverse events are identified, these will be reported to the principal investigator and if deemed clinically significant and related to the agent, we will arrange for an in-person assessment.

Part 1

1. Surgery with patients under anesthesia is anticipated to have a duration of 4-6h and will be conducted per routine.

2. An arterial line will be placed to monitor vital signs and allow for sequential blood draws throughout the case. Arterial lines are placed in select patients undergoing prostatectomy, but this is not standard for all cases. Patients will be informed that this monitoring will be additional to the standard anesthesia anticipated for such cases and will be reviewed in the informed consent document.

3. Blood will be drawn at baseline and at 15, 30, 120, 240, 480 minutes (+/- 10 min), 12 hours (+/- 1h), 18 hours (+/- 2h), and 24 hours (+/- 2h) after administration for

pharmacokinetics analysis in all patients. In addition, a minimum of 3 patients in each dose cohort will also have blood drawn at 48 hours (+/- 24h) and 72 hours (+/- 24h) after administration for pharmacokinetics analysis.

4. The obturator nerve will be used as the reference nerve, and intensity of fluorescence will be recorded. The obturator was selected as the reference nerve for the following reasons: it is a nerve structure universally encountered during all pelvic lymph node dissections, owing to its anatomically consistent location; fat, vasculature, lymphatics, and muscle structures are adjacent and can serve as background tissues for fluorescent intensity comparisons; it is a myelinated structure that would be anticipated to fluoresce after administration of Illuminare-1 in humans; and it has fluoresced in all animal models used to date when assessed. Fluorescence will be graded on a 5-point scale.

Intensity of Fluorescence Subjective Scale:

1 = no fluorescence of obturator nerve

2 = no difference in contrast between background enhancement and reference nerve

3 = minimal contrast between obturator nerve fluorescence and background normal tissues

4 = moderate contrast between obturator nerve fluorescence and background normal tissues

5 = maximal contrast between obturator nerve fluorescence and background normal tissues

5. Surgeon subjective assessments of obturator nerve fluorescent intensity will be measured at 30 minutes (± 5 minutes) after intravenous administration of Illuminare-1 and every 20 minutes (± 5 minutes) thereafter, as long as the measurement of fluorescent intensity will not negatively affect the ongoing portion of the surgical procedure. If the fluorescent intensity of the nerve cannot be assessed at a specific time point because it would disrupt the flow of the surgical case, this will be recorded, and the assessment will be made at the next scheduled time point. The anticipated time required to make a subjective assessment of the fluorescent intensity of the obturator nerve, on the basis of preclinical observations, is between 30 and 60 seconds per assessment. We anticipate this may add an additional 7-15 minutes to the procedure with the patient under anesthesia. One surgeon observer will record the obturator nerve to background fluorescent intensity at each time point.

6. Simultaneously, video recordings will be captured for *post hoc* analysis under white light and blue light for objective, quantitative assessment of NMR. These data will be used to inform future hardware and software optimization for later studies.

7. Simultaneously, 24 hour ECGs will be collected via Holter monitor for *post hoc* analysis.

8. Resection of the nerve bundles and histologic confirmation are not mandatory and are not expected as part of the protocol. They will be performed only on structures that would normally be removed as part of the standard of care surgery the patient will receive. Patients with high-risk disease features (high PSA, abnormal digital rectal exam, Gleason sum 8 or higher disease) or MRI features concerning for extracapsular extension of disease are more likely to have a wider resection of neurovascular tissues to achieve a negative surgical margin. We anticipate the wide resection of neurovascular bundles will be infrequently performed—in ≤5 patients total—during the dose-escalation part of the study; this number will depend on the cohort of patients enrolled and the clinical risk factors associated with the individual patients' prostate cancer. There may be a higher number of patients with these disease characteristics enrolled during the expansion phase of the protocol.

9. Adverse events will be recorded and reported as necessary for the first 24 h after injection or until discharge and at the first postoperative visit (10 ± 5 days). Adverse events will also be collected by phone at 30 (± 5) days, and in person at the follow-up visit corresponding to the first postoperative PSA lab assessment, which typically occurs approximately 7 weeks after surgery (45 ± 10 days). Blood will be drawn at each in person follow-up visit for potential future research purposes under MSK IRB#06-107 or MSK IRB #12-245.

Part 2

1. In this dose-expansion part of the study, we will continue to assess the safety of Illuminare-1 at the dose selected following completion of Part 1.

2. In this part of the study, we will evaluate, in addition to the obturator nerve, the neurovascular bundles before opening the endopelvic fascia and during the nerve-sparing portion of the prostatectomy (if performed as part of the standard of care surgery). We will look at the neurovascular bundles after removal of the prostate to see whether a fluorescent signal consistent with nerve structures within the neurovascular bundle can be identified. One surgeon observer will record whether presumed nerve structures are visualized under blue-light conditions (Yes vs. No); if nerve structures are seen, the confidence level of this assessment will be recorded (graded scale of 1-4, as described previously). The extent of surgery performed and the degree of nerve sparing that was undertaken will not be altered on the basis of the fluorescence of the

presumed location of the neurovascular bundles. This will be optionally performed during Part 1 if possible. The sensitivity and specificity of this agent's ability to accurately localize the small nerves within the neurovascular bundles have not been established, and no surgical decisions will be made on the basis of the fluorescence of structures in the periprostatic regions.

3. If the surgeon identifies a presumed nerve structure that is destined to be resected with the prostate as part of the standard of care surgery being performed, this presumed fluorescent nerve structure may be tagged with a fine suture for *ex vivo* harvesting for histologic analysis for the presence or absence of nerve structures. If the surgeon identifies a structure that is destined to be resected as part of the standard surgery being performed and the surgeon believes that structure could possibly be a nerve although it is not fluorescing, this ambiguous structure may be differently tagged with a fine suture for *ex vivo* harvesting for histologic analysis for the presence or absence of nerve structures. In either case, the surgical specimen will be examined *ex vivo* remote from the operative field under blue light, and additional nerve structures not initially tagged *in vivo* by the operating surgeon will be tagged for histologic analysis. The tagged structures presumed to be nerve and adjacent tissues with similar visible appearance (i.e., vascular or lymphatic structures running in parallel to the fluorescent, tagged structure) will be assessed histologically for the presence or absence of nerve tissue.

   The obturator nerve will not be tagged, harvested, or manipulated in any manner other than what would be done as a normal part of the standard surgery being performed.

4. Adverse events will be recorded and reported as necessary for the first 24 hours after injection or until discharge; at the first postoperative visit (10 ± 5 days); by telephone at 30 (± 5) days, and at the follow-up visit corresponding to the first postoperative PSA lab assessment, which typically occurs approximately 7 weeks after surgery (45 ± 10 days). If any adverse symptoms are reported during the telephone follow up at 30 (± 5) days, the primary investigator will be notified and the patient will be scheduled for an in-person assessment. One tube of blood (<10 cc) will be drawn at the two in-person follow up visits for potential future research purposes under MSK IRB#06-107 or MSK#12-245.

5.0 THERAPEUTIC/DIAGNOSTIC AGENTS & NON-THERAPEUTIC ASSESSMENTS

5.1 Investigational Agent

The investigational agent is Illuminare-1 (HCl salt) formulated in isotonic 12.5% Captisol/sterile water for injection.

Illuminare-1 is being developed at MSK in collaboration with Illuminare Biotechnologies. MSK will be the sponsor of the first-in-human phase I clinical trial. Illuminare Biotechnologies holds the license for this agent and intends to further develop Illuminare-1 toward a marketing application.

Chemical Name: 2-(4-(4-(4-(4-Aminostyryl)-3-methoxystyryl)phenylsulfonyl)piperazin-1-yl) ethanol hydrochloride

Pharmacological Class: Fluorescent small-molecule myelin-binding agent

Structural Formula:

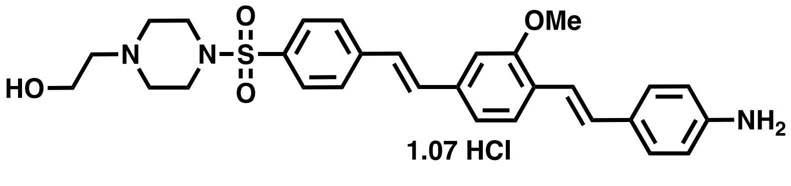

1.07 HCl

Chemical Formula: $C_{29}H_{33}N_3O_4S$. 1.07 HCl
Molecular Weight: 519.66 + (1.07 X 36.46) = 558.67

Physical Characteristics: Yellow to light orange colored solution

Formulation: Illuminare-1 is formulated in 12.5% Captisol/sterile water for injection, in clear vials containing 10.5 mL per vial at a concentration of 4.0 mg/mL for intravenous injection.

Storage: Illuminare-1 is a liquid formulation that is stored in the freezer at -20ºC. The evening before the procedure, the vial is moved to a 4ºC refrigerator overnight to allow ample time for thawing. Illuminare-1 will be kept from light during storage.

Source of Supply: The clinical drug will be dispensed and inventoried by the MSK pharmacy. The pharmacy will be provided instructions for the preparation of study drug and dosing administration procedures. Illuminare-1 HCl salt is manufactured by the MSK Organic Synthesis Core Facility and vialed by the Clinical Grade Production Core under good manufacturing practices appropriate for phase I clinical trials.

5.2 Investigational Device

This clinical trial will be conducted using an investigational device that consists of the FDA-approved Karl Storz D-Light C photodynamic diagnostic (PDD) rigid system, with a minor modification to a standard 10-mm 0-degree lens surgical laparoscope.

The Storz PDD system is indicated for photodynamic blue-light cystoscopy as an adjunct to white-light cystoscopy for the detection of non-muscle-invasive papillary cancer of the bladder

in patients suspected or known to have the lesion on the basis of a prior cystoscopy when used in combination with the optical imaging drug Cysview (hexaminolevulinate hydrochloride). Cysview will not be used in this study. Since Cysview and Illuminare-1 are fluorescent under similar optical conditions, the Storz PDD system will be used as the hardware system in this phase 1 study due to its proven safety profile, prior FDA approval in a similar application, and our extensive experience utilizing the system in the operative setting.

The Storz PDD system is routinely used by the Urology Service at MSK for endoscopic resection of bladder tumors. It includes a 4-mm cystoscope (sized to access the bladder) with an optical filter located on the eyepiece of the PDD telescope, to provide contrast between fluorescing and non-fluorescing tissue.

To obtain an appropriate field of view for abdominal laparoscopy in this study, the FDA-approved 10-mm Hopkins II telescope and Karl Storz ALA snap-on optical filter (rather than embedded) will be used (P/N 20100034).

The eyepiece contains a fluorescein blue filter, identical to the filter embedded in the eyepiece of the 4-mm rigid cystoscope used for standard blue-light cystoscopy. The filter material is also the same as in the 4-mm rigid cystoscope and was acquired through collaboration with engineers at Karl Storz Endoscopy. It is sterilizable, fits all standard 10-mm laparoscopy lenses, and has been used successfully in animal labs by the teams at MSK, Karl Storz, and Intuitive Surgical Systems. There will be no physical contact between the snap-on lens/camera system and the patient's body.

With the exception of the 10-mm laparoscope and filter, the premarket approval (PMA 050027)–approved Karl Storz PDD system will be used without modification; the investigational device essentially duplicates the approved cystoscope. The ALA snap-on filter is not FDA approved at this time. The eyepiece does not come in contact with the patient during the procedure. After the procedure, the eye piece will be cleaned per MSK's SOP for device disinfection.

When used in tandem with the Intuitive da Vinci robotic surgery platform, the fluorescent camera system will be placed through the assistant port only during fluorescence measurements.

The system has 3 modes of operation: an attenuated white-light mode (Mode 1) for use during fluorescence examinations, a PDD or blue-light mode (Mode 2) for identification of fluorescent images, and a high-intensity white-light mode (Mode 3) for normal endoscopic examinations when the PDD system is not in use. Videos will be captured on the standard MSK recording device.

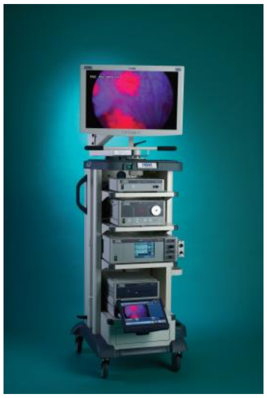

Figure 2. The Karl Storz D-Light C PDD rigid system

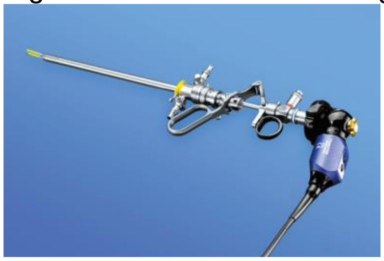

Figure 3. Four-millimeter PDD cystoscope included in the Storz PDD system

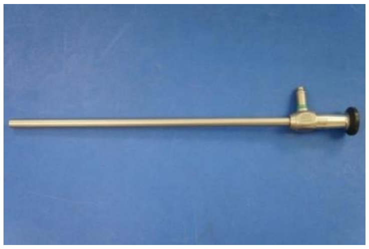

Figure 4. Ten-millimeter Hopkins II telescope used in the Illuminare-1 clinical trial

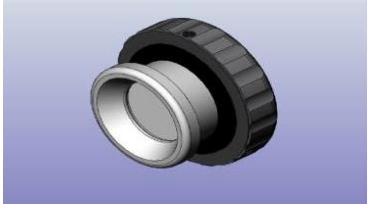

Figure 5. Karl Storz ALA snap-on optical filter

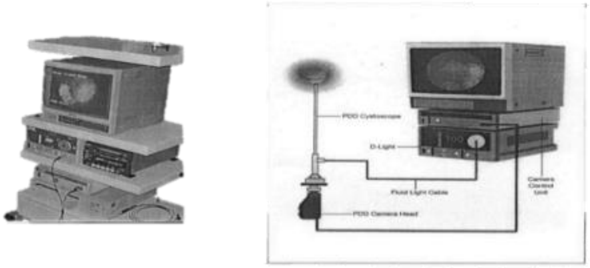

Figure 6. Laparoscope, filter, and camera interface

Table 1. List of approved and investigational device components

**FDA-approved equipment system components**	**Method of use in phase I, compared with approved system**	**If modified, rationale for modification**	**Risk assessment**	
Karl Storz D-Light C PDD light source	Identical	Not applicable	Approved; no added risk	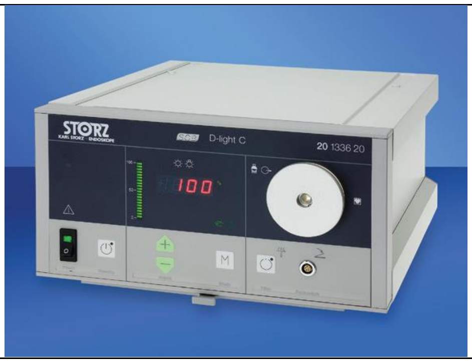

TRICAM SL II camera control unit	Identical	Not applicable	Approved; no added risk	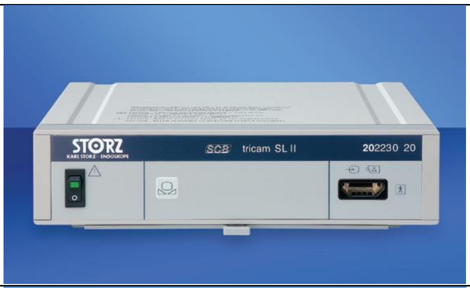
TRICAM PDD 3-Chip camera head	Identical	Not applicable	Approved; no added risk	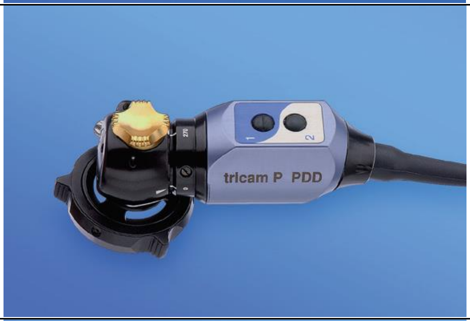
Fluid light cable	Identical	Not applicable	Approved; no added risk	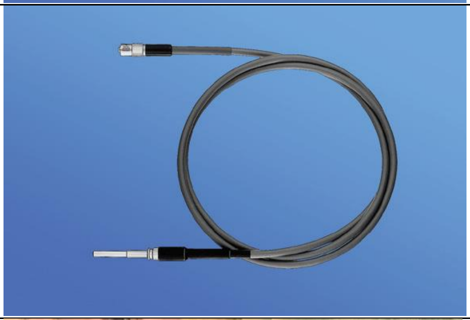
Hopkins II 10-mm telescope	Modified	The approved PDD system uses a 4-mm cystoscope that has a filter embedded in the eyepiece portion of the instrument. The 4-mm cystoscope lens does not offer a wide enough field of view to be useful in abdominal laparoscopy; therefore, a standard FDA-approved 10-mm laparoscope with an attachable, rather than glued-on, filter will be used for laparoscopic visualization. The filter is the same as the one used for the cystoscope, was acquired through collaboration with Karl Storz Endoscopy and	Approved; no added risk	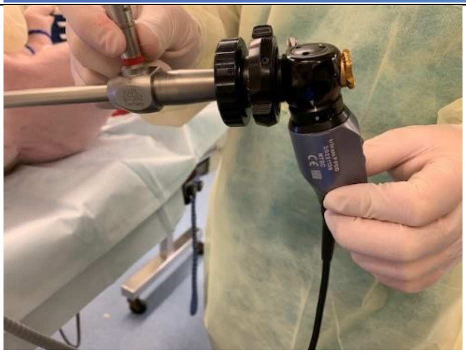

		their engineers, is sterilizable, fits all standard 10-mm Hopkins laparoscopy lenses, and has been used by the teams at MSK, Karl Storz, and Intuitive Surgical Systems successfully in animal labs.		
**Unapproved equipment system component**				
Optical snap-on filter	Modified	As above; no patient contact	Low risk	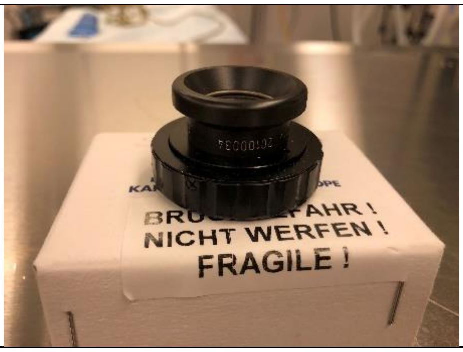

Device Sterilization

The device parts that will be in contact with sterile regions in the operating room will be a standard 10-mm laparoscope, which will be sterilized according to current MSK equipment protocols. All parts will be sterilized according to the manufacturer's specifications. Other than the laparoscope, there will be no physical contact between the snap-on lens and the camera system with the patient's body. The operating personnel can operate the device and change its position during the operation, as with any handheld laparoscopic lens.

6.0 CRITERIA FOR PARTICIPANT ELIGIBILITY

This first-in-human study will be a phase I study of up to 50 patients with adenocarcinoma of the prostate undergoing minimally invasive radical prostatectomy. All patients enrolled in the study will undergo surgery as normally performed to current standards of care at MSK. The planned surgery and extent of dissection will not be altered based upon the fluorescent imaging of structures visualized during the case. Patients will be managed perioperatively according to existing MSK pathways developed in compliance with current recommendations and guidelines.

6.1 Participant Inclusion Criteria

Patients must be:

- 18 years or older and capable of signing their own consent form
- Scheduled for minimally invasive radical prostatectomy with pelvic lymph node dissection

6.2 Participant Exclusion Criteria

Exclusion from the study will occur for patients with the following:

- Prior pelvic surgery or pelvic radiation therapy
- Known central nervous or peripheral nervous system disease or insult (including neurodegenerative diseases), current use of neurotoxic medications, or use of cytotoxic chemotherapy with known neurotoxicity within 1 month
- Exposure to investigational agents in the immediate 30 days before or 15 days after study drug administration
- Patients with significant renal dysfunction (<60 mL/min as assessed by the Cockcroft Gault calculation for creatinine clearance)
- Clinically significant hepatic/liver impairment (liver function tests more than 2x institutional normal upper limit)
- Exposure to phototoxic drugs such as St. John's wort, griseofulvin, thiazide diuretics, sulfonylureas, phenothiazines, sulphonamides, quinolones, and tetracyclines, should be avoided. If safe to discontinue use for a limited time, patients may participate on this trial but will discontinue the use of the interfering drug for 5 half-lives before Illuminare-1 injection to permit full washout, as well as for 48 hours after administration of Illuminare-1.

7.0 RECRUITMENT PLAN

7.1 Research Participant Registration

Patients with prostate cancer scheduled to undergo surgery at MSK will be recruited from the practices of the Division of Urology. The study will be introduced to prospective patients seen by the investigator and coinvestigators. Routine standard of care surgery will be performed, and enrollment in the trial will not alter the surgery planned to be performed. The study will be available to the public, and the details of the inclusion criteria, exclusion criteria, and study design will be posted at www.clinicaltrials.gov.

Potential research subjects will be identified by a member of the patient's treatment team, the protocol investigator, or research team at MSKCC. If the investigator is a member of the treatment team, s/he will screen their patient's medical records for suitable research study participants and discuss the study and their potential for enrolling in the research study. Potential subjects contacted by their treating physician will be referred to the investigator/research staff of the study. If the potential participant has not already consented

to MSKs biospecimen study, 06-107 or 12-245 they will be approached and consented for both studies concurrently.

The principal investigator may also screen the medical records of patients scheduled to undergo radical prostatectomy with whom he does not have a treatment relationship for the limited purpose of identifying patients who would be eligible to enroll in the study and to record appropriate contact information in order to approach these patients regarding the possibility of enrolling in the study.

During the initial conversation between the investigator/research staff and the patient, the patient may be asked to provide certain health information that is necessary to the recruitment and enrollment process. The investigator/research staff may also review portions of their medical records at MSKCC in order to further assess eligibility. They will use the information provided by the patient and/or medical record to confirm that the patient is eligible and to contact the patient regarding study enrollment. If the patient turns out to be ineligible for the research study, the research staff will destroy all information collected on the patient during the initial conversation and medical records review, except for any information that must be maintained for screening log purposes.

In most cases, the initial contact with the prospective subject will be conducted either by the treatment team, investigator, or the research staff working in consultation with the treatment team. The recruitment process outlined presents no more than minimal risk to the privacy of the patients who are screened, and minimal PHI will be maintained as part of a screening log. For these reasons, we seek a (partial) limited waiver of authorization for the purposes of (1) reviewing medical records to identify potential research subjects and to obtain information relevant to the enrollment process; (2) conversing with patients regarding possible enrollment; (3) handling of PHI contained within those records and provided by the potential subjects; and (4) maintaining information in a screening log of patients approached (if applicable).

Patients will be screened on the basis of disease type and surgery status. Informed consent will be obtained before surgery. Candidate patients will be allowed time to consider the study, read the informed consent document at their convenience, and discuss it with family and others, as desired. Once the patient is ready to provide informed consent, this will occur in-person, with the subject providing written informed consent. Obtaining consent over the phone is not permitted.

Estimated accrual: On the basis of clinical volumes of similar cases and previous clinical trials conducted at MSK, the estimated accrual is 2 to 4 patients per month overall. We anticipate accrual of the potential maximum of 50 patients will take less than 18 months.

7.2 Randomization

Not applicable.

8.0 INFORMED CONSENT PROCEDURES

Before protocol-specified procedures are carried out, consenting professionals will explain full details of the protocol and study procedures as well as the risks involved to participants prior to their inclusion in the study. Participants will also be informed that they are free to withdraw from the study at any time. All participants must sign an IRB/PB-approved consent form indicating their consent to participate. This consent form meets the requirements of the Code of Federal Regulations and the Institutional Review Board/Privacy Board of this Center. The consent form will include the following:

1. The nature and objectives, potential risks and benefits of the intended study.
2. The length of study and the likely follow-up required.
3. Alternatives to the proposed study. (This will include available standard and investigational therapies. In addition, patients will be offered an option of supportive care for therapeutic studies.)
4. The name of the investigator(s) responsible for the protocol.
5. The right of the participant to accept or refuse study interventions/interactions and to withdraw from participation at any time.

Before any protocol-specific procedures can be carried out, the consenting professional will fully explain the aspects of patient privacy concerning research specific information. In addition to signing the IRB Informed Consent, all patients must agree to the Research Authorization component of the informed consent form.

Each participant and consenting professional will sign the consent form. The participant must receive a copy of the signed informed consent form.

9.0 PRE-TREATMENT/INTERVENTION

This protocol includes all pretreatment evaluations currently included in the clinical care standards for patients undergoing surgery at MSK. These include:

- Routine history and physical examination, to include documentation of any comorbidities, medications (including complementary and alternative medications), surgical history, family history, social history (alcohol and tobacco use), height, body weight, and Karnofsky performance status within 30 days of surgery
- Multiparametric prostate MRI before surgery
- Preoperative laboratory investigations: complete blood cell count, basic chemistry panel, urinalysis, and urine culture if indicated within 30 days of surgery

Patients will undergo baseline assessments of cognitive and neurologic function by use of the following validated instruments: the Beijers questionnaire for CTC grading of peripheral neuropathy (Appendix B) and the Neuropathy Impairment Scale–Lower Limbs (Appendix A). These same validated instruments will be used at the postoperative time points listed above;

if a change from baseline is identified, a full neurologic assessment will be performed to determine whether the change is considered to be related to the drug.

10.0 TREATMENT/INTERVENTION PLAN

1. The operating team will consist of surgeons on faculty in the MSK Department of Surgery in the Division of Urology. The appropriate lines and monitors will be placed by the anesthesiology team and general anesthesia will be induced. Invasive monitoring will be at the discretion of the anesthesia team.

2. The procedures will be performed with the patient under general anesthesia in accordance with standard procedures. Intravenous fluid use during surgery will be provided by the anesthesiology team as clinically indicated. Hypovolemic resuscitation will be avoided. The total volume of fluids provided (typically 2000-2500cc of crystalloids during the case), as well as estimated blood loss, will be recorded.

3. The patient will receive a single intravenous dose of Illuminare-1 over 5 min at the assigned dose level approximately 30 minutes before anticipated real-time need for intraoperative nerve visualization, which corresponds to the start time of the pelvic lymph node dissection portion of the surgery. The precise timing of administration of Illuminare-1 will be at the discretion of the operating surgeon, according to the procedure being performed.

4. In the preoperative area, Holter monitors will be placed on patients for 24-hour research ECG data collection. We plan on ECG extractions at the following timepoints:
   a. 3 baseline timepoints preoperatively
   b. 0.25, 0.5, 2, 4, 8, 12, 24 hours postdose to align with the PK timepoints

   For these ECG extractions, the patient will be supine in a comfortable resting position for ~10 minutes, the first 5 minutes to allow the heart rate and autonomic tone to stabilize, and the second 5 minutes for the ECG ‘extraction window’. Exact timepoints will be recorded.

5. Blood will be drawn at baseline, 15, 30, 120, 240, 480 minutes (all +/- 10min), 12 hours (+/- 1h), 18 hours (+/- 2h), and 24 hours (+/- 2h) after injection for pharmacokinetics evaluation in all patients. In addition, a minimum of 3 patients in each dose cohort will also have blood drawn at 48 hours (+/- 24h) and 72 hours (+/- 24h) after administration for pharmacokinetics evaluation. Blood draws for PK evaluation will be done after the ECG extraction window.

6. Surgery will be performed in accordance with current standards of care at MSK. No additional laparoscopic trocars other than those routinely used will be required. The standard 10mm laparoscope with the attached fluorescent camera system will be placed through the assistant port during the brief portions of the case when visualization under blue-light and white-light conditions is needed. The surgical approach will be at the discretion of the surgeon. None of

the techniques used during the surgery are considered to be experimental and all are considered standard therapeutic options for patients undergoing radical prostatectomy.

7. The duration of the surgical procedure will vary depending on the type of approach and the patient's clinical characteristics. The duration of the surgical procedure will be noted and images and videos will be captured.

8. Assessment of Illuminare-1 fluorescence will be assessed as described in section 4.2.

   All tissue harvesting will be performed under the supervision of pathology faculty. For ethical reasons, the protocol does not authorize the excision of additional fluorescent deposits detected *in vivo* outside of the planned resection regions by use of the fluorescent camera system. However, excision of such tissues may be performed at the discretion of the operating surgeon if he/she deems it clinically necessary, safe, and in compliance with current standards of care to do so.

9. The entire intraoperative procedure will be video recorded with the collection of both conventional and fluorescent images and will be archived according to institutional guidelines. Videos will be used for *post hoc* collection of fluorescence imaging data (to avoid interrupting the surgical procedure).

10. It is estimated that visualization of the pertinent anatomy using the fluorescent camera system for the purposes of this study will add no more than 15 min to the total operative time. The actual time necessary for fluorescent imaging will be recorded as part of the study.

11. Patients exposed to a photosensitizing agent may experience a phototoxic skin reaction, such as a sunburn, when exposed to excessive ultraviolet light or phototoxic drugs. As a precaution and because the half-life of the agent is approximately 4 hours, we will avoid administration of phototoxic drugs such as St. John's wort, griseofulvin, thiazide diuretics, sulfonylureas, phenothiazines, sulphonamides, quinolones, and tetracyclines for 24 hours before and after administration of Illuminare-1. These medications are uncommonly administered during the standard preoperative and postoperative pathway for patients undergoing minimally invasive radical prostatectomy.

12. Patients will be evaluated prior to discharge on postoperative day 1 and repeat neurologic examinations performed at baseline will be performed. Patient will also receive a CTN telephone call within 2 days of surgery for toxicity evaluation.

13. Patients will be evaluated at the postoperative visit on POD 10 (+/- 5). Repeat neurologic examinations performed at baseline will be performed. One tube of blood will be drawn for future research purposes under MSK IRB#06-107 or MSK IRB#12-245.

14. Patients will be evaluated at a postoperative visit on POD 30 (+/- 5) with a telephone follow-up. Patients are not routinely seen between the day 10 visit and the first PSA lab assessment so a telephone visit to assess for toxicity will be performed and if necessary, an in-person visit will be coordinated if there is clinical concern.

15. Patients will be evaluated at the postoperative visit on POD 45(+/- 10) corresponding to the first PSA blood level assessment. Repeat neurologic examinations performed at baseline will be performed. One tube of blood will be drawn for future research purposes under MSK IRB#06-107 or MSK IRB #12-245.

Timeline of Interventions and Assessments

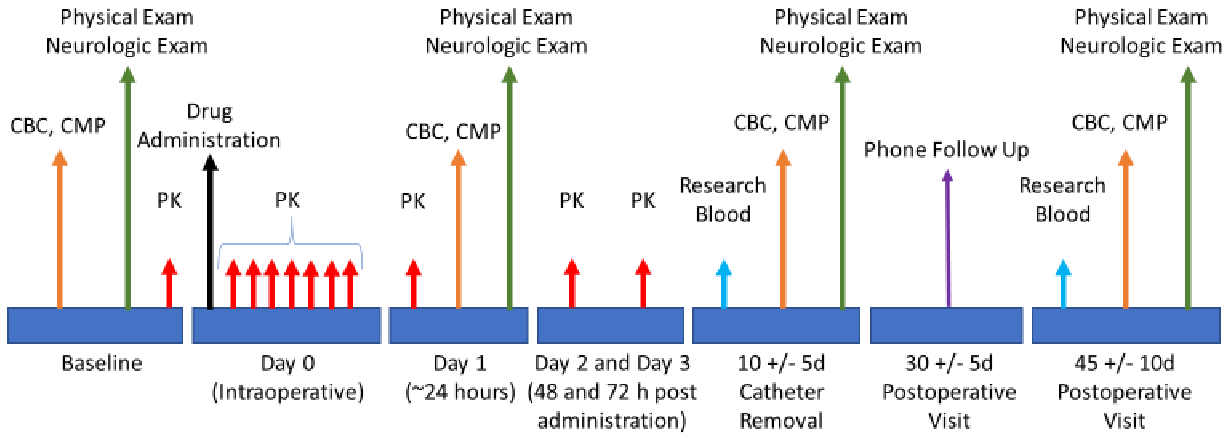

11.0 EVALUATION DURING TREATMENT/INTERVENTION

	Preop/Eligibility	Perioperative	Postsurgery (<24 h)	Postsurgery (48 h and 72 h post injection)	Postsurgery (day 10 ± 5)	Phone Follow-Up (30± 5 days)	Follow-Up Visit 45 ± 10 days)
Informed consent	X						
Concomitant medication check	X		X				
History and physical exam*	X		X		X		X
Multiparametric prostate MRI*	X						
Vital signs*	X	X	X		X		X
Karnofsky performance status*	X				X		X
Standard presurgical testing*	X						

CBC (standard) and comprehensive metabolic panel*	X		X		X		X
Urinalysis	X						
Urine culture (if indicated)	X						
ECG	X		X				
Research ECG via 24-hour Holter monitor †		X	X				
Adverse events** CTCAE 5.0			X		X	X	X
Neurologic assessment	X		X		X		X
Subjective fluorescence assessment of obturator nerve		X					
Subjective fluorescence assessment of neurovascular bundles		X					
Blood sampling for pharmacokinetics ***		X	X	X			
PSA lab assessment*							X
Blood for future research purposes (under 06-107 or 12-245)					X		X
Ex vivo tissue H&E			X				
Video evaluation (duration of surgery, NMR)		X					

*Standard of care evaluations.

**Serious adverse events will be reported from the time of informed consent to the end of study evaluation (45 +/- 10 days). Adverse events considered to be related to the drug that are grade ≥2 will be reported from the time of Illuminare-1 administration to the end of study evaluation. Adverse events from the time of informed consent to Illuminare-1 administration will be recorded in the medical history. Postsurgery (<24 hr) AE evaluation will occur via a CTN phone call within 2 days of surgery.

***Blood will be drawn at baseline, 15, 30, 120, 240, 480 minutes (all +/- 10min), 12 hours (+/- 1h), 18 hours (+/- 2h), and 24 hours (+/- 2h) after injection for pharmacokinetics evaluation in all patients. In addition, a minimum of 3 patients in each dose cohort will also have blood drawn at 48 hours (+/- 24h) and 72 hours (+/- 24h) after administration for pharmacokinetics evaluation.

† ECG extraction timepoints: 3 baseline timepoints preoperatively; 0.25, 0.5, 2, 4, 8, 12 and 24 hours post-dose to align with PK timepoints. Patients to be comfortably resting in a supine position for ~10 minutes, the first 5 minutes to allow the heart rate and autonomic tone to stabilize, and the second 5 minutes for the ECG 'extraction window'. Exact timepoints will be recorded.

Investigational Agent Evaluations

Subjects will remain in-patient after administration of Illuminare-1 for 24 h before discharge. During this period, the patients will be monitored for adverse events according to CTCAE 5.0.

At 10 (± 5) days, patients will be assessed during the routinely scheduled postoperative visit which may coincide with catheter removal. Patients will also be questioned about adverse events at 30 (± 5) days via telephone, and at the first postoperative PSA lab assessment, which typically occurs around 7 weeks after surgery (45 ± 10 days).

The pharmacokinetics of Illuminare-1 will be evaluated from blood drawn at baseline, 15, 30, 120, 240, 480 minutes (all +/- 10 min), 12 hours (+/- 1h), 18 hours (+/- 2h), and 24 hours (+/- 2h) after injection in all patients. In addition, a minimum of 3 patients in each dose cohort will also have blood drawn at 48 hours (+/- 24h) and 72 hours (+/- 24h) after administration for pharmacokinetics evaluation. A minimum of 3mL whole blood will be collected into 5 mL EDTA purple top tubes. Handling instructions (e.g., sample processing, centrifugation, storage conditions) will be provided to the processing laboratory via SOP prior to sample collection. One tube of blood (<10cc) will be drawn and stored for potential future research investigations at in-person follow-up visits 10 days (± 5 days) post-surgery and the first PSA blood draw (45 ± 10 days). The samples for future research will be taken under MSK IRB#06-107 or MSK IRB#12-245. All pharmacokinetics analyses will be performed using Phoenix WinNonLin (version 8.1) and a noncompartmental approach consistent with the intravenous route of administration. Pharmacokinetics model selection will be based on a visual inspection of goodness-of-fit plots (observations vs. predictions, residuals and weighted residuals vs. predictions). The assay values (concentrations vs. times) measured using a validated LC-MS method will be used to calculate half-life, volume of distribution, clearance, individual-specific elimination rate constant (Ke), time to maximum concentration (Tmax), maximum concentration (Cmax), and area under the concentration–time curve (AUC) values. Pharmacokinetic analyses will be conducted at MSK by core facilities and the statistician will not perform these.

Pharmacokinetic blood sampling at 48 hours (+/-24 hours) and 72 hours (+/-24hours) post injection as well as blood sampling for future research purposes at the Day 10 (+/-5 days) and Day 45 (+/- 10 days) follow up visits may be obtained by The National Phlebotomy Provider Network (NPPN) when patients are not able to travel to an MSKCC facility or the MSKCC facility is closed. NPPN is a national medical specimen collection, processing, and delivery service. NPPN provides mobile medical specimen collection, processing, and delivery services to medical diagnostic laboratories, biotech companies, research organizations, medical practices, independent physician and non-physician practitioners, and individual patients under a physician’s order.

NPPN will collect and process the specimen in accordance with specified protocols from the MSKCC laboratory. Additionally, NPPN will ship, deliver, or courier the specimens to the MSKCC laboratory in accordance with specified protocols from the MSKCC laboratory. In order

to coordinate these collections, the NPPN will have access to patient's name, address, and phone number.

Neurologic assessments will be performed at presurgery, before discharge from the hospital after surgery (approximately 24 h), and at in-person follow-up visits (10 ± 5 days and 45 ± 10 days after surgery) using validated surveys. The battery of neurologic tests will be administered by the research team in coordination with and under the supervision of our co-investigators in the Neurology Division.

12.0 CRITERIA FOR REMOVAL FROM STUDY

Patients will be withdrawn from the study if they express a desire to do so, if it is determined to be in the patient's best interest to do so, or if they do not undergo initiation of their surgical procedure. Given that patient involvement occurs only during the operation itself, we anticipate that very few (if any) patients will withdraw after consent. Patients who are not evaluable for either the safety or response endpoints will be excluded from the analysis and replaced.

Additional reasons for patient removal from the study include but are not limited to:

- Adverse events
- Abnormal laboratory values
- Protocol deviation
- Withdrawal of consent
- Death

Patients who withdraw from the trial due to either personal reasons or adverse events related to the study drug will be described in detail. Symptoms and, if applicable, follow-up testing related to the adverse event will be recorded during the hospital stay and at each follow up visit. All patients in this trial will undergo surgery, with a minimum hospital stay of 1 day; therefore, no patient should be lost to short-term follow-up. All patients will return at 10 (+/- 5) and 45 (+/- 10) days for postoperative visits. In the unlikely event that patients are lost to follow-up, they will be contacted by telephone.

13.0 CRITERIA FOR OUTCOME ASSESSMENT AND ENDPOINT EVALUABILITY

13.1 Criteria for Therapeutic Response/Outcome Assessment

This study is not designed to assess a therapeutic response.

13.2 Criteria for Study Endpoint Evaluability

Protocol participants will become evaluable for the safety analysis at the time of study drug administration. Patients who are removed within the DLT period due to reasons not associated with the trial primary endpoint will be replaced.

Five patients will be observed at each dose level. If > 20% of patients within a cohort experience a grade 2 or higher adverse event that is considered attributable to the drug and clinically significant, dose escalation will be stopped at that level, and the prior dose level will be considered the MTD. If the desired response endpoint (sustained grade 4 fluorescence for 90 minutes or longer) is achieved in 3 patients at a single dose level, another 5 patients will be enrolled at this dose level. If sustained subjective grade 4 fluorescence is again observed in 3 of the next 5 patients, escalation to an incremental dose (not to exceed +0.5mg/kg) may be assessed in 5 additional patients to measure any potential improvement over the prior dose. Improvement is defined as sustained visualization of the obturator at grade 5 observed in 3/5 patients at the incrementally higher dose. If no improvement is seen at the incremental dose, the previous dose would be considered an acceptable imaging dose. If improvement is observed, another 5 patients will be enrolled at this dose. If sustained subjective grade 4 fluorescence is not observed in 3 of this second group of 5 patients, this will not be a sufficient dose, and escalation to an incremental dose (not to exceed +0.5mg/kg) will be tested. Once an acceptable imaging dose level has been selected, an expansion cohort will be undertaken, which will not exceed 20 patients. The expansion cohort may be stopped at any point prior to reaching the 20th subject if it is deemed that adequate safety and efficacy information has been collected.

14.0 BIOSTATISTICS

This is a phase I dose-escalation trial with twin endpoints of safety and fluorescent intensity at each dose level. Dose levels of the agent will be administered starting at 0.25mg/kg, with increases in dose between cohorts of no more than 0.5 mg/kg until a maximum dose of 2.25mg/kg is reached, if needed. Dose escalation rules are as follows:

1) Enter the first 5 patients at a given dose.
    a. If > 1 patient has toxicity (defined as grade ≥2 toxicity attributable to the agent), prior dose level is MTD.
    b. If ≥ 3 patients have response (defined as sustained grade 4 fluorescence for 90 minutes or longer) and <2 have toxicity, add an additional 5 patients.
    c. If neither condition (a) nor (b) is met, dose escalate
2) For the second cohort of 5 patients
    a. If >2 patients have a toxicity at the dose level, including the first five patients, prior dose level is MTD.
    b. If ≥ 6 patients in total have response (including the first five patients in the cohort) and toxicity rate is ≤20%, add up to 20 additional patients
    c. If neither condition (a) nor (b) is met, dose escalate

3) For the expansion cohort of up to 20 patients.
   a. If at any point > 20% patients have a toxicity at the dose level, including the first 10 patients, prior dose level is MTD.

The effective dose will be chosen as the highest tolerable dose at which, in 2 consecutive cohorts of 5 patients, 3 patients respond. The probability that this criterion will be met is ~80% if the true response probability is 75%, 25% if the true response probability is ~50%, and ~10% if the true response probability is 40%. The effective dose must be proven safe such that < 2 patients in the first 5 patients have a toxicity and at no point as the number of patients is escalated to up to 20 does the proportion of patients with toxicity exceed 20%. The probability of declaring a dose safe, assessed by simulation study, is 5%, 21%, 72% and 95% for true toxicity rates of 5%, 10%, 20% and 30% respectively.

Secondary Objectives:

Pharmacokinetic analysis will be conducted by MSK. Change from baseline for the Neuropathy Impairment Scale will be described at the three postoperative timepoints by giving the quartiles and top 10% highest scores. Surgeon subjective assessments of obturator nerve fluorescent intensity will be calculated using general estimating equations with time, dose and a time × dose interaction term using a binary endpoint of adequate fluorescence (score of 4 or 5). This analysis will be repeated for blue fluorescence vs. white-light by comparing the coefficient and standard error thereof for dose and time × dose interaction. No quantitative analyses are planned for *in vivo* and/or *ex vivo* fluorescent signal of presumed nerve tissue with corresponding histologic evaluation in patients undergoing a wider resection.

15.0 TOXICITIES/RISKS/SIDE EFFECTS

If an adverse event after administration of the agent occurs, the clinical signs, symptoms, laboratory assessments, and other observations will be recorded. The following definitions will be used to assess causality:

**Not related:**	The clinical adverse event is definitely unrelated to the study treatment (e.g., does not follow a reasonable temporal sequence from study treatment, present prior to receiving study medication, etc.)
**Unlikely**	The study treatment is not likely to have had reasonable association with the observed experience; however, relationship cannot be definitely excluded.
**Possible**	The connection with study treatment appears unlikely, but cannot be excluded with certainty (e.g., follows a reasonable temporal sequence from drug administration, may be related to known

	characteristics of the patients' clinical state or other modes of therapy administered to the patient, etc.).
**Probable**	The clinical adverse event appears related to the study treatment with a high degree of certainty (e.g., follows a reasonable temporal sequence from drug administration and abates upon discontinuation of the drug, cannot be reasonably explained by known characteristics of the patient's clinical state or other modes of therapy administered to the patient, etc.).
**Definite**	The event follows a reasonable temporal sequence from the time of drug administration, and follows a known response pattern to the study drug, cannot be reasonably explained by other factors such as the patient's condition, therapeutic interventions or concomitant drugs; AND occurs immediately following study drug administration, improves on stopping the drug, or reappears on re-exposure.

The intensity of an adverse event will be graded according to the NCI Common Toxicity Criteria v 5.0. Adverse events that cannot be graded using the NCI Common Toxicity Criteria will be graded as mild (asymptomatic), moderate (symptomatic but not interfering significantly with function) or severe (causing significant interference with function).

If the causality of the event is scored as “possibly”, “probably”, or “definitely” related to administration of the investigational agent, the event will also be scored according to the Clavien-Dindo classification. Grade 2 or higher adverse events will be considered clinically significant events for study purposes.

Subjects will remain in the hospital after administration of Illuminare-1 for approximately 24 hours before discharge. During this period, the patients will be monitored for adverse events according to CTCAE 5.0 and undergo neurologic examination in addition to the two neurologic instruments for assessing peripheral nerve function, the Beijers questionnaire for CTC grading of peripheral neuropathy (Appendix B) and the Neuropathy Impairment Scale–Lower Limbs (Appendix A). These validated instruments will be used at the postoperative time points listed in the next paragraph below; if a change from baseline is identified, a full neurologic assessment will be performed to determine whether the change is considered to be related to the drug. These neurologic assessments are not part of the routine standard of care evaluations performed for patients undergoing minimally invasive radical prostatectomy and are being done as part of the safety profile assessment of the agent.

Following surgery, all adverse events will be evaluated at 10 (± 5) days, at 30 (± 5) days (additional telephone call primarily for toxicity assessment), and at 45 (± 10) days after surgery (day of first PSA lab assessment and postoperative visit). We will record the number of subjects in each cohort who experience a DLT after receiving Illuminare-1. The entire follow-up period for patients on study will last no more than 45 (± 10) days after surgery.

DLTs will be defined as follows, using NCI-CTCAE 5.0:

1. Hematologic Toxicity
   a. Study treatment–related febrile neutropenia
   b. Grade ≥2 neutropenia lasting >7 days
   c. Study treatment–related grade ≥2 thrombocytopenia or bleeding associated with grade ≥2 thrombocytopenia

2. Nonhematologic Toxicity
   a. Study treatment–related grade ≥2 nonhematological toxicity
   b. Any drug-related death

15.1 Serious Adverse Event (SAE) Reporting

An adverse event is considered serious if it results in ANY of the following outcomes:

- Death
- A life-threatening adverse event
- An adverse event that results in inpatient hospitalization or prolongation of existing hospitalization
- A persistent or significant incapacity or substantial disruption of the ability to conduct normal life functions
- A congenital anomaly/birth defect
- Important Medical Events (IME) that may not result in death, be life threatening, or require hospitalization may be considered serious when, based upon medical judgment, they may jeopardize the patient or participant and may require medical or surgical intervention to prevent one of the outcomes listed in this definition

Note: Hospital admission for a planned procedure/disease treatment is not considered an SAE.

SAE reporting is required as soon as the participant starts investigational treatment/intervention. SAE reporting is required for 30-days after the participant's last investigational treatment/intervention. Any event that occur after the 30-day period that is unexpected and at least possibly related to protocol treatment must be reported.

Please note: Any SAE that occurs prior to the start of investigational treatment/intervention and is related to a screening test or procedure (i.e., a screening biopsy) must be reported.

All SAEs must be submitted in PIMS. If an SAE requires submission to the HRPP office per IRB SOP RR-408 ‘Reporting of Serious Adverse Events’, the SAE report must be submitted within 5 calendar days of the event. All other SAEs must be submitted within 30 calendar days of the event.

The report should contain the following information:

- The date the adverse event occurred
- The adverse event
- The grade of the event
- Relationship of the adverse event to the treatment(s)
- If the AE was expected
- Detailed text that includes the following
    - An explanation of how the AE was handled
    - A description of the participant’s condition
    - Indication if the participant remains on the study
- If an amendment will need to be made to the protocol and/or consent form
- If the SAE is an Unanticipated Problem

15.1.1 Toxicity Assessment and Stopping Criteria

This is a first in human study of this agent and no clinically significant side effects are expected based upon pre-clinical data and toxicity assessments across multiple animal models. We did note some reversible glomerular changes in the kidneys at high doses (>13 x what will be administered in this trial), so we plan to follow renal function as a safety parameter in addition to monitoring for other AEs and SAEs. Additionally, we recognize that this agent binds to myelin during its short half-life and that neurologic assessments are necessary and appropriate stopping criteria have been developed. Patients will be closely monitored while admitted to the hospital and laboratory values obtained after will be reviewed. Abnormal laboratory findings (e.g., clinical chemistry, hematology, urinalysis) or other abnormal assessments (e.g., EKGs, vital signs) that are judged by the Investigator as clinically significant will be recorded as AEs or SAEs if they meet the definition of an AE or SAE.

15.1.1.1 Renal Toxicity Stopping Criteria

Clinical chemistry threshold stopping criteria have been designed to assure subject safety. When subjects meet the following clinical chemistry criteria, additional testing will be performed, and the subject monitored until chemistries resolve, stabilize, or return to baseline values. The subject will not be withdrawn from the study since no further administration of the agent will be performed (it is a one-time use agent and will have already been given) and the data regarding fluorescent intensity of the nerve of interest will already have been obtained.

Patients with significant renal dysfunction (<60 mL/min as assessed by the Cockcroft Gault calculation for creatinine clearance) will not be enrolled on the trial as per initial exclusion criteria. Creatinine values will be obtained at 4 hours and 14 hours after surgery or as per standard of care after surgery. If creatinine assessments after administration of the investigational agent identify a doubling in baseline creatinine or a GFR < 50 ml/min/1.73m2), the patient will be assessed to determine whether other likely etiologies for the renal dysfunction are present, such as acute blood loss, hypotension, administration of known nephrotoxic medications, etc. The primary investigator will coordinate these assessments with the primary team to determine whether the change in renal function is clinically significant and attributable to the investigational agent. Repeat renal function assessments will be made twice weekly until creatinine levels resolve, stabilize or return to within baseline values and the patient will be referred to a nephrologist for consultation.

15.1.1.2. Neurological Stopping Criteria

Neurological threshold stopping criteria have been designed to assure subject safety. When subjects meet the following neurological criteria, additional testing will be performed and the subject monitored until abnormal parameters resolve, stabilize or return to baseline values.

Neurological Assessments include the Beijers questionnaire for CTC grading of peripheral neuropathy (CTCAE version 4) (Appendix B) and the Neuropathy Impairment Scale–Lower Limbs (Appendix A) (Dyck PJ, Litchy WJ, Lehman KA, et al: Variables influencing neuropathic endpoints: the Rochester Diabetic Neuropathy Study of Healthy Subjects. Neurology 1995; e45 (6): 1115-21.).

Patients with Grade 2 or higher neurotoxicity such as confusion, ataxia, weakness, or numbness require neurologic assessment, which will include an examination with a neurologic specialist for a detailed neurologic examination, an MRI of the brain and spine, and nerve conduction and electromyography.

15.1.1.3. Injection Site Reaction (ISR) Stopping Criteria

- Any grade 3 signs of hypersensitivity or injection site reaction attributable to the agent.

15.1.1.4. Additional Stopping Criteria

Individual Subjects will be withdrawn if they show poor tolerability with respect to signs and symptoms, clinically significant changes in laboratory parameters or vital signs.

Safety parameters from a given cohort will be fully assessed before dose escalation and escalation will proceed according to the dose escalation criteria outlined in the protocol. Any trends towards treatment-related changes will be fully evaluated.

Any subject with clinically significant changes in safety parameters not listed above or significant AEs thought to be treatment-related will be withdrawn from study treatment and

monitored until recovery. A full assessment by the primary investigator and/or the safety monitoring board will determine whether it is safe and ethical for the subject to continue.

15.2 External SAE Reporting

Not Applicable

16.0 PROTECTION OF HUMAN PARTICIPANTS

16.1 Privacy

MSK's Privacy Office may allow the use and disclosure of protected health information pursuant to a completed and signed Research Authorization form. The use and disclosure of protected health information will be limited to the individuals/entities described in the Research Authorization form. A Research Authorization form must be approved by the IRB and Privacy Board (IRB/PB).

The consent indicates that individualized de identified information collected for the purposes of this study may be shared with other qualified researchers, including Illuminare and Gray Optics. Only researchers who have received approval from MSK will be allowed to access this information which will not include protected health information, such as the participant's name, except for dates. It is also stated in the Research Authorization that their research data may be shared with others at the time of study publication.

This study will be carried out in compliance with the protocol and the principles of Good Clinical Practice, as described by standard operating procedures and:

- US 21 Code of Federal Regulations dealing with clinical studies (including parts 50 and 56 concerning informed consent and IRB regulations)
- Declaration of Helsinki and amendments, concerning medical research in humans

Benefits and Risks: The use of surgical imaging to improve nerve visualization during surgery is not routine, owing to a dearth of specific targeted tracers and a lack of data on safety, toxicity, feasibility, efficacy, and reproducibility. Previous preclinical and preliminary studies have suggested that fluorescent imaging–guided nerve identification holds potential benefits for patients undergoing surgery. Therefore, we do not believe that the imaging aspects of this trial pose any additional risks, compared with those faced by patients undergoing a surgery off-study.

Toxicities/Side Effects: This is a first-in-human study of this agent. Toxicity has been assessed according to standard preclinical evaluations, and adverse outcomes have not been associated with the doses of Illuminare-1 to be used in this study.

Alternatives/Options for Treatment: The alternative to participation in the trial would be to undergo surgery in accordance with the surgeon's standard practice without having fluorescent imaging–guided inspection and assessment of the surgical field. No other aspect of patient care would differ.

Financial Costs/Burdens: The subjects will not be compensated for their participation, and there are no costs involved with participation.

Privacy and Confidentiality: Every effort will be made to keep the study records private. No identifiers will be used in any reports or publications resulting from the study; however, the data will be used in the interests of ongoing research.

Voluntary Nature of the Study: Participation is entirely voluntary. All aspects of patient care and monitoring will be unaffected by whether the patient chooses to participate in the study.

16.2 Data Management

Final data sets for publication are required to be locked and stored centrally for potential future access requests from outside entities.

Data will be managed using Medidata. This is the primary data collection instrument for this study. Digital video of the procedure will be recorded and permanently stored on the secure, password-protected server, according to the Joint Commission on the Accreditation of Healthcare Organizations guidelines.

The Indication for CTC Grading of Peripheral Neuropathy scale will be completed by patients in REDCap. REDCap, Research Electronic Data Capture, is an open source platform that allows for the collection of research data in a secure manner over a web based interface. Usage of the platform is contingent on an open source license. The platform was developed by Vanderbilt University which MSK has a standing agreement with to allow the usage of REDCap for academic/research purposes.

For this protocol, electronic data entry forms may be completed online by study staff. Electronic participant responses will also be collected either by sending participants a direct link or having the participants fill out the electronic survey on site.

Data will be housed in the Memorial Sloan Kettering Cancer Center's (MSKCC) New Jersey data center. REDCap has been approved by MSKCC's Information Security to store PHI. The MSKCC Information Systems group is responsible for applying all operating system patches and security updates to the REDCap servers. All connections to REDCap utilize encrypted (SSL-based) connections to ensure data is protected. The server is backed up nightly in the event that disaster recovery would be necessary and system would need to be rolled back. Members of the Clinical Research Administration supporting the REDCap software will have access to REDCap projects for the purpose to ensuring the proper functioning of the database and the overall software system.

Permissions to the database for internal users will be managed by the REDCap project manager or study staff. User access to the data is contingent on those a part of the study team and data sharing agreements in place with third party entities if applicable. Project managers are responsible for regularly auditing these permissions to ensure changes in staff are reflected appropriately.

REDCap has the ability maintain an audit trail of changes to the database providing a timestamp as well as the user making the update. In addition, a data resolution module offers the ability of opening and closing queries optionally requiring justification when data is being updated. Permission roles for data resolution are integrated in REDCap. Comprehensive system logs are also maintained of user activity and when changes to the database are made.

At the conclusion of the study, deidentified data will be shared with Illuminare via a secure FTP site established by MSK IT. They will be receiving data as a part of our collaboration on this project.

16.3 Quality Assurance

Original study case report forms will be scanned and stored permanently in the patient's electronic medical record (EMR). These forms will therefore be available to rectify any possible transcription errors in the process of data transfer to the electronic database. Monthly registration reports will be generated to monitor patient accrual and completeness of registration data. Routine data quality reports will be generated to assess missing data and inconsistencies. Accrual rates and extent and accuracy of evaluations will be monitored throughout the study period. Potential problems will be brought to the attention of the study team for discussion and action.

16.4 Data and Safety Monitoring

The Data and Safety Monitoring Plan utilized for this study must align with the MSK DSM Plan, where applicable.

The Data and Safety Monitoring (DSM) Plans at Memorial Sloan Kettering were approved by the National Cancer Institute in August 2018. The plans address the new policies set forth by the NCI in the document entitled "Policy of the National Cancer Institute for Data and Safety Monitoring of Clinical Trials."

There are several different mechanisms by which clinical studies are monitored for data, safety and quality. At a departmental/PI level there exists procedures for quality control by the research team(s). Institutional processes in place for quality assurance include protocol monitoring, compliance and data verification audits, staff education on clinical research QA and two institutional committees that are responsible for monitoring the activities of our clinical trials programs. The committees: *Data and Safety Monitoring Committee (DSMC)* for Phase I

and II clinical trials, and the *Data and Safety Monitoring Board (DSMB)* for Phase III clinical trials, report to the Center's Research Council and Institutional Review Board.

During the protocol development and review process, each protocol will be assessed for its level of risk and degree of monitoring required. Every type of protocol (e.g., NIH sponsored, in-house sponsored, industrial sponsored, NCI cooperative group, etc.) will be addressed, and the monitoring will be established at the time of protocol activation.

17.0 REFERENCES

1. Gibbs-Strauss S, Nasr K, Fish K, et al. Nerve-highlighting fluorescent contrast agents for image-guided surgery. Mol Imaging. 2011;10(2):91-101.

2. Bajaj A, LaPlante NE, Cotero VE, al. Identification of the protein target of myelin-binding ligands by immunohistochemistry and biochemical analyses. J Histochem Cytochem. 2013;61:19-30.

3. Walsh EM, Cole D, Tipirneni KE, et al. Fluorescence imaging of nerves during surgery. Ann Surg. 2019;270:69-76.

4. Cotero VE, Kimm SY, Siclovan TM, et al. Improved intraoperative visualization of nerves through a myelin-binding fluorophore and dual-mode laparoscopic imaging. PLoS ONE. 2015;10(6):e0130276

5. Roberts DW, Valdes BS, Harris BT, et al. Coregistered fluorescence-enhanced tumor resection of malignant glioma: relationships between ∂-aminolevulinic acid-induced protoporphyin IX fluorescence, magnetic resonance imaging enhancement, and neureopathological parameters. J Neurosurg. 2011; 114: 595-603.

6. Couccia D, Fandino J, Fujioka, et al. Intraoperative 5-aminolevulinic-acid-induced fluorescence in meningiomas. Aca Neuorchir. 2010: 152:1781-1719.

7. Stummer W, Stocker S, Wagner S, et al. Intraoperative detection of malignant gliomas by 5-aminolevulinic acid-induced porphyrin fluorescence. Neurosurgery. 1998;42(3):518-25.

8. Dean LW, Recabal P, Dylov DV, et al. V12-05 intraoperative nerve visualization with GE3126. J Urol. 2018:199:e1237-e8.

18.0 APPENDICES

Appendix A: Neurologic Evaluation Surveys

Appendix B: Beijers Questionnaire Scoring

Appendix C: NPPN Instructions Future Research Collections

Appendix D NPPN Instructions PK Collections
