# Supplementary material for: Rizedisben in Minimally Invasive Surgery: A Nonrandomized Clinical Trial
Source: JAMA Surg. 2025 Jul 2;160(8):875–83. doi: 10.1001/jamasurg.2025.1987 (PMC12224043; doi:10.1001/jamasurg.2025.1987)
Supplement: Supplement 2. — Data sharing statement [file jamasurg-e251987-s002.pdf]

Data Sharing Statement

Gold. Rizedisben in Minimally Invasive Surgery. *JAMA Surg*. Published July 02, 2025.
doi:10.1001/jamasurg.2025.1987

Data
Additional Information: Clinicaltrial.gov Identifier: NCT04983862
Data available: No
